# Supplementary material for: Investigation of the Detailed AMPylated Reaction Mechanism for the Huntingtin Yeast-Interacting Protein E Enzyme HYPE
Source: Int J Mol Sci. 2021 Jun 29;22(13):6999. doi: 10.3390/ijms22136999 (PMC8267892; doi:10.3390/ijms22136999)
Supplement: Supplementary file 1 [file ijms-22-06999-s001.zip › ijms-1264138-supplementary.pdf]

Supplementary Materials

Investigation of the detailed AMPylated reaction mechanism for the Huntingtin yeast-interacting protein E enzyme HYPE

Meili Liu, Zhe Huai, Hongwei Tan, Guangju Chen

Table of Contents

Figure S1. Electrostatic potential (ESP) on the molecular vdW surface of 6-coordinated (a), 5-coordinated (5), and 4-coordinated(c) in the reactant state. All the ESP calculations are performed at the level of B3LYP/6-311++G(d,p)..1

Figure S2. (a) RMSD analysis of the whole complexed protein. (b) RMSD analysis of ATP around 5 Å in complexes...2

Figure S3. The root-mean-squared fluctuation (RMSF) of C-α atoms of complex during conventional MD simulations...3

Figure S4. Number of Hydrogen Bonds. ATP forms hydrogen bonds involving ATP with surrounding proteins, which evolve over time. 4

Figure S5. The details of HYPE and BiP protein docking are supplemented to explain the building model process. HYPE protein selected molecule A. BiP protein selected the part of molecule B' that included from the terminal to the critical part containing Thr518 (the part within the dashed line.)...6

Figure S6. Superimposition of the average structure of the final 10ns for 70ns in three MD simulations. (a) Cartoon state. (b) Sticks state. (c) Partial enlarged view of the area indicated by the structures surrounding ATP and Arg374 residue in (b).. 7

Table S1. The electrostatic potential (ESP) of Model-1 with 6-coordinated (at B3LYP/6-311++G (d, p) level)). Unit of B-factor field (i.e. ESP) is kcal/mol. 8

Table S2. The electrostatic potential (ESP) of Model-2 with 5-coordinated (at B3LYP/6-311++G (d, p) level))...12

Table S3. The electrostatic potential (ESP) of Model-3 with 4-coordinated (at B3LYP/6-311++G (d, p) level)). 15

Table S4. Cartesian coordinates of 6-coordinated...18

Table S5. Cartesian coordinates of 5-coordinated...32

Table S6. Cartesian coordinates of 4-coordinated AMPylation. 45

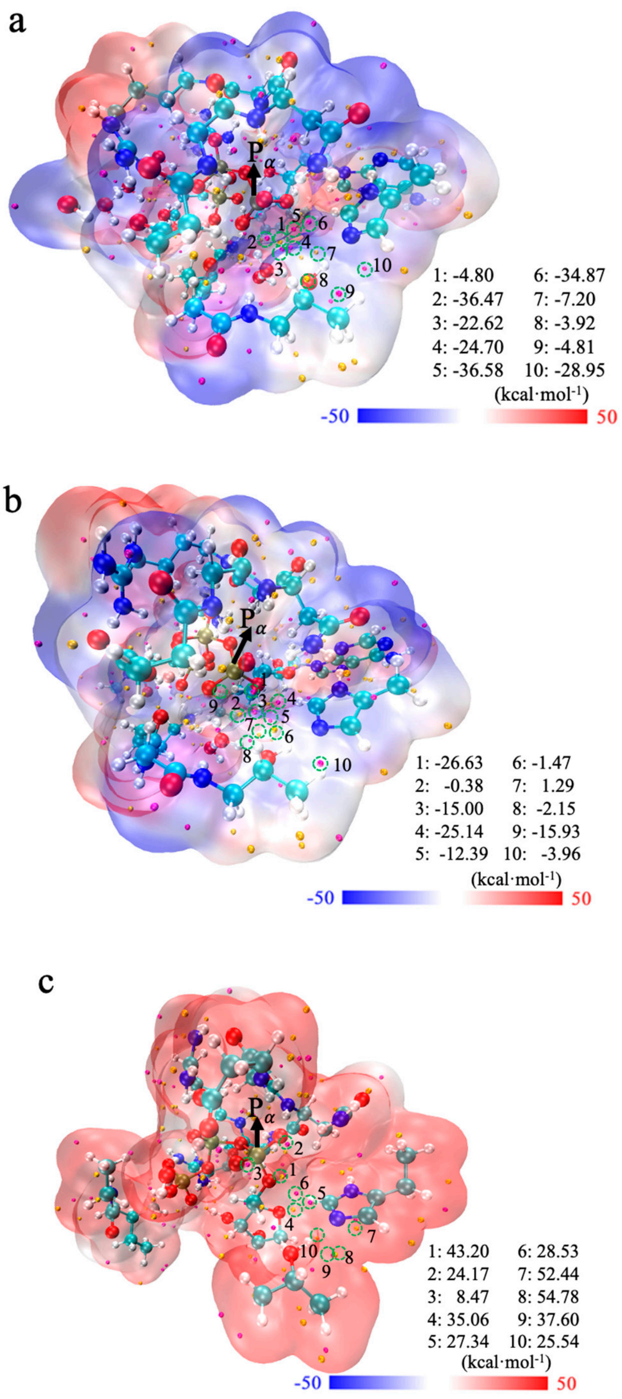

Figure S1. Electrostatic potential (ESP) on the molecular vdW surface of 6-coordinated (a), 5-coordinated (5), and 4-coordinated(c) in the reactant state. All the ESP calculations are performed at the level of B3LYP/6-311++G(d,p). Yellow and magenta spheres denote the surface local maxima and minima of the ESP values. Local maxima and minima of the ESP values in the most important regions are numbered and corresponding values are also provided.

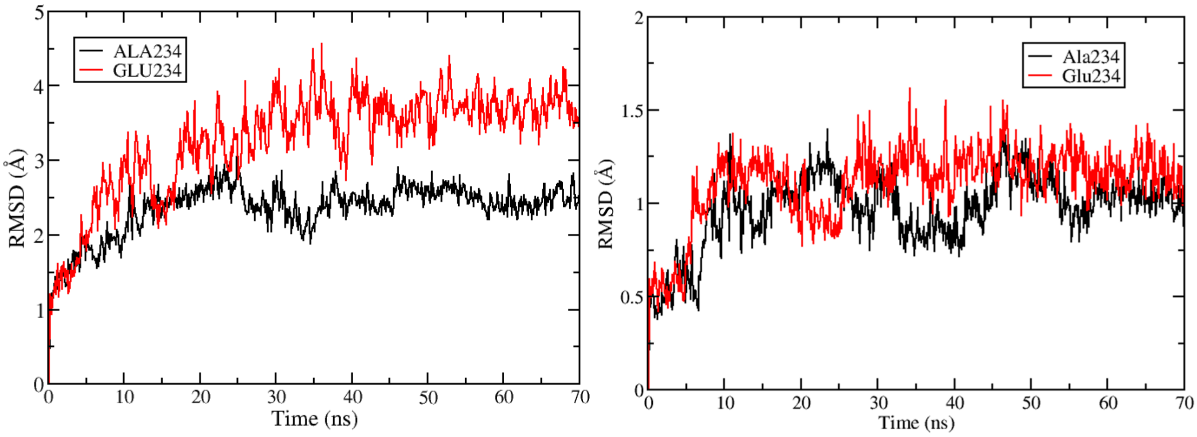

Figure S2. (a) RMSD analysis of the whole complexed protein. (b) RMSD analysis of ATP around 5 Å in complexes.

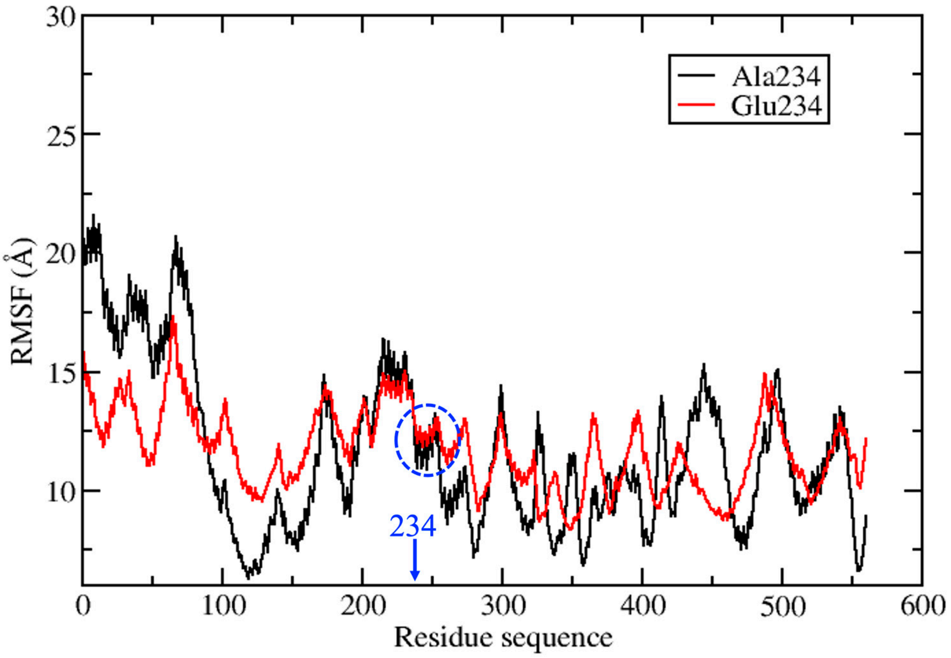

Figure S3. The root-mean-squared fluctuation (RMSF) of C-α atoms of complex during conventional MD simulations.

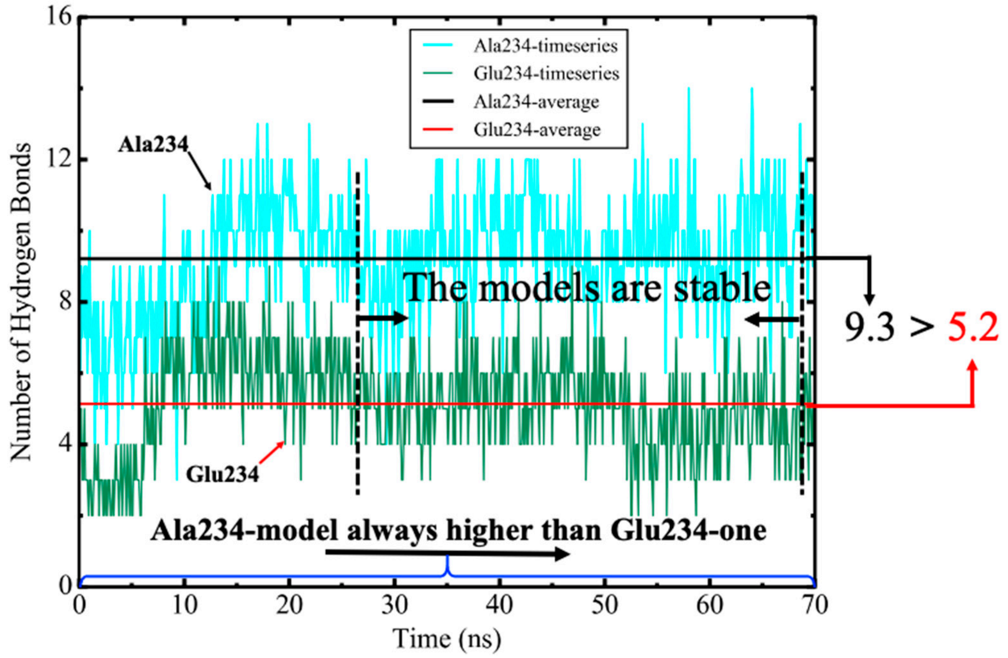

Figure S4. Number of Hydrogen Bonds. ATP forms hydrogen bonds involving ATP with surrounding proteins, which evolve over time.

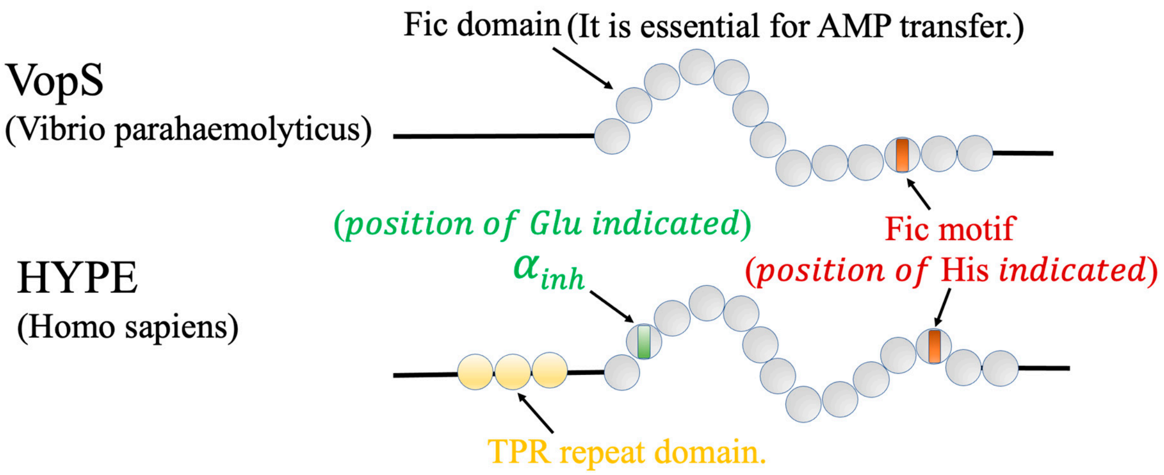

Scheme S1. Schematic representations of the domain organization of the fic-domain-containing AMPylases VopS (Vibrio parahaemolyticus), and Huntingtin yeast-interacting protein E (HYPE) (Homo sapiens). Key amino acids of the active site motif (His) and inhibitory motif (Glu) are highlighted in red and green, respectively.

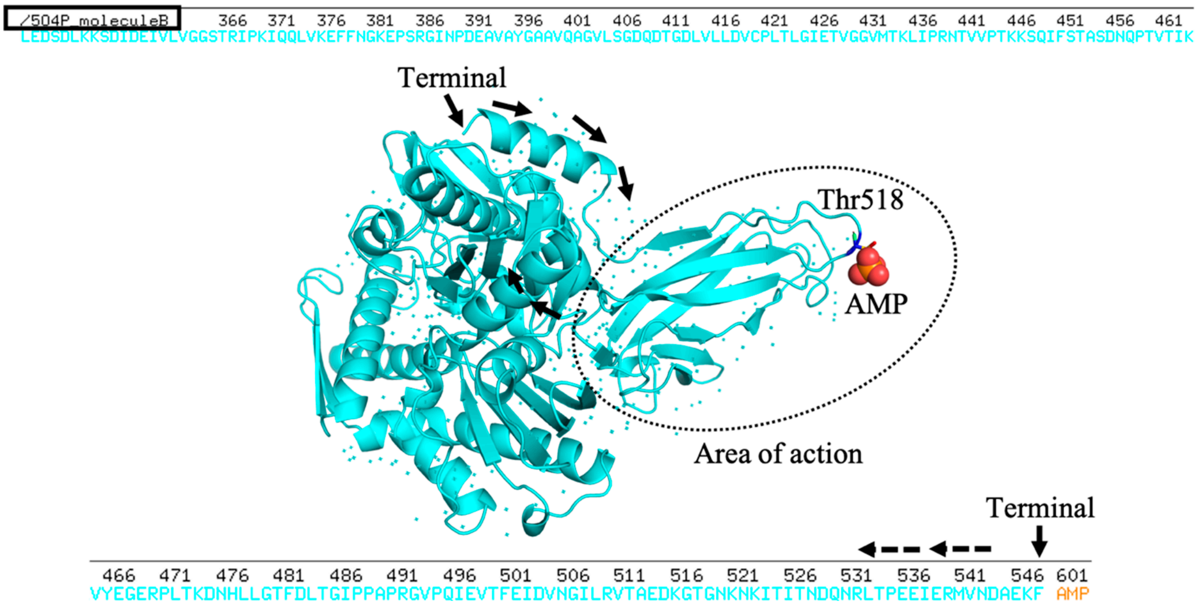

Figure S5. The details of HYPE and BiP protein docking are supplemented to explain the building model process. HYPE protein selected molecule A. BiP protein selected the part of molecule B' that included from the terminal to the critical part containing Thr518 (the part within the dashed line.)

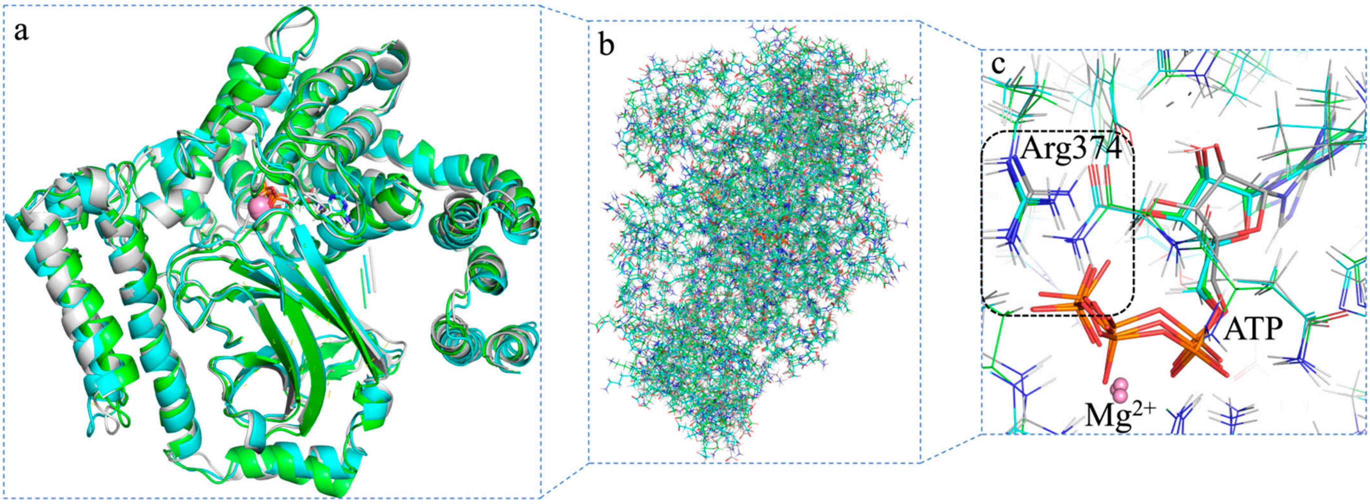

Figure S6. Superimposition of the average structure of the final 10ns for 70ns in three MD simulations. (a) Cartoon state. (b) Sticks state. (c) Partial enlarged view of the area indicated by the structures surrounding ATP and Arg374 residue in (b). Mg^{2+} is shown in ball. ATP and Arg374 are shown in sticks.

Table S1. The electrostatic potential (ESP) of Model-1 with 6-coordinated (at B3LYP/6-311++G (d, p) level)). Unit of B-factor field (i.e. ESP) is kcal/mol.

HETATM	1	C	MOL	A	1	-7.444	-0.178	-8.881	1.00	21.36	C
HETATM	2	C	MOL	A	1	-6.940	1.534	0.924	1.00	23.15	C
HETATM	3	C	MOL	A	1	-6.574	-2.399	-1.339	1.00	43.26	C
HETATM	4	C	MOL	A	1	-6.487	-1.431	-7.581	1.00	21.10	C
HETATM	5	C	MOL	A	1	-6.396	0.572	5.285	1.00	56.96	C
HETATM	6	C	MOL	A	1	-6.353	3.983	-7.136	1.00	54.53	C
HETATM	7	C	MOL	A	1	-6.072	5.961	1.808	1.00	22.59	C
HETATM	8	C	MOL	A	1	-6.001	1.477	-3.450	1.00	11.39	C
HETATM	9	C	MOL	A	1	-5.907	1.451	-9.852	1.00	25.76	C
HETATM	10	C	MOL	A	1	-5.221	-0.167	-0.519	1.00	22.88	C
HETATM	11	C	MOL	A	1	-5.196	2.247	-2.324	1.00	15.75	C
HETATM	12	C	MOL	A	1	-5.126	-1.335	-4.006	1.00	13.73	C
HETATM	13	C	MOL	A	1	-5.099	6.076	4.358	1.00	13.78	C
HETATM	14	C	MOL	A	1	-5.009	-4.789	4.329	1.00	-4.48	C
HETATM	15	C	MOL	A	1	-5.019	6.934	-1.207	1.00	60.49	C
HETATM	16	C	MOL	A	1	-3.891	0.490	5.599	1.00	5.59	C
HETATM	17	C	MOL	A	1	-3.777	-0.936	-4.004	1.00	11.13	C
HETATM	18	C	MOL	A	1	-3.745	1.556	-6.943	1.00	15.00	C
HETATM	19	C	MOL	A	1	-3.689	-4.823	1.315	1.00	6.02	C
HETATM	20	C	MOL	A	1	-3.479	-5.578	-5.629	1.00	19.24	C
HETATM	21	C	MOL	A	1	-3.507	1.396	2.900	1.00	2.15	C
HETATM	22	C	MOL	A	1	-3.188	-1.142	2.919	1.00	-0.49	C
HETATM	23	C	MOL	A	1	-2.747	3.268	-0.434	1.00	9.73	C
HETATM	24	C	MOL	A	1	-2.418	-7.344	-2.553	1.00	49.61	C
HETATM	25	C	MOL	A	1	-2.119	0.266	-4.235	1.00	21.28	C
HETATM	26	C	MOL	A	1	-2.028	6.209	-2.581	1.00	12.07	C
HETATM	27	C	MOL	A	1	-1.625	3.751	-2.580	1.00	14.48	C
HETATM	28	C	MOL	A	1	-1.156	-5.232	5.770	1.00	-0.23	C
HETATM	29	C	MOL	A	1	-1.212	-5.134	2.132	1.00	-2.36	C
HETATM	30	C	MOL	A	1	-0.932	0.325	6.612	1.00	-1.29	C
HETATM	31	C	MOL	A	1	-0.866	6.758	-0.451	1.00	14.52	C
HETATM	32	C	MOL	A	1	-0.739	-3.070	5.781	1.00	-4.56	C
HETATM	33	C	MOL	A	1	-0.537	-2.367	-2.921	1.00	10.27	C
HETATM	34	C	MOL	A	1	-0.271	6.622	3.704	1.00	0.56	C
HETATM	35	C	MOL	A	1	-0.207	-1.137	-7.697	1.00	26.37	C
HETATM	36	C	MOL	A	1	-0.219	3.173	-4.644	1.00	8.67	C
HETATM	37	C	MOL	A	1	0.119	-4.434	3.460	1.00	-9.22	C
HETATM	38	C	MOL	A	1	0.502	5.092	-0.041	1.00	25.70	C
HETATM	39	C	MOL	A	1	0.599	-1.816	-5.762	1.00	33.58	C
HETATM	40	C	MOL	A	1	0.591	-0.862	5.685	1.00	-3.21	C
HETATM	41	C	MOL	A	1	0.829	6.925	3.459	1.00	-0.23	C
HETATM	42	C	MOL	A	1	0.984	3.226	0.659	1.00	4.36	C
HETATM	43	C	MOL	A	1	0.985	5.333	-0.593	1.00	29.79	C
HETATM	44	C	MOL	A	1	1.069	1.449	7.261	1.00	-6.58	C
HETATM	45	C	MOL	A	1	1.202	-0.467	-4.574	1.00	30.98	C
HETATM	46	C	MOL	A	1	1.497	3.361	1.270	1.00	-0.95	C
HETATM	47	C	MOL	A	1	1.626	0.419	6.404	1.00	-5.21	C
HETATM	48	C	MOL	A	1	1.884	-2.606	0.545	1.00	-4.80	C
HETATM	49	C	MOL	A	1	2.175	1.919	2.185	1.00	-5.17	C

HETATM	50	C	MOL	A	1	2.326	4.695	2.499	1.00	-10.23	C
HETATM	51	C	MOL	A	1	2.506	-5.134	3.553	1.00	-3.92	C
HETATM	52	C	MOL	A	1	2.572	-8.229	1.791	1.00	-0.67	C
HETATM	53	C	MOL	A	1	2.889	-7.341	-1.439	1.00	4.30	C
HETATM	54	C	MOL	A	1	2.920	3.141	3.297	1.00	-8.21	C
HETATM	55	C	MOL	A	1	3.009	-3.399	-4.918	1.00	49.75	C
HETATM	56	C	MOL	A	1	3.120	-0.077	2.798	1.00	-9.71	C
HETATM	57	C	MOL	A	1	3.163	-2.808	-0.892	1.00	-1.30	C
HETATM	58	C	MOL	A	1	3.460	1.056	10.019	1.00	15.87	C
HETATM	59	C	MOL	A	1	3.470	-7.819	3.694	1.00	1.26	C
HETATM	60	C	MOL	A	1	3.542	-2.892	5.599	1.00	20.01	C
HETATM	61	C	MOL	A	1	3.524	0.609	-2.617	1.00	17.79	C
HETATM	62	C	MOL	A	1	3.765	-2.756	-0.122	1.00	-7.20	C
HETATM	63	C	MOL	A	1	4.412	4.388	-4.238	1.00	17.32	C
HETATM	64	C	MOL	A	1	4.523	0.871	3.773	1.00	-2.87	C
HETATM	65	C	MOL	A	1	4.611	2.867	4.072	1.00	-5.54	C
HETATM	66	C	MOL	A	1	4.702	3.264	10.218	1.00	12.84	C
HETATM	67	C	MOL	A	1	4.677	3.586	-3.646	1.00	25.57	C
HETATM	68	C	MOL	A	1	5.032	-7.559	0.249	1.00	2.33	C
HETATM	69	C	MOL	A	1	5.309	5.608	-6.667	1.00	12.16	C
HETATM	70	C	MOL	A	1	5.388	4.735	-0.793	1.00	10.55	C
HETATM	71	C	MOL	A	1	5.674	-4.832	4.263	1.00	7.74	C
HETATM	72	C	MOL	A	1	5.993	0.258	0.596	1.00	1.84	C
HETATM	73	C	MOL	A	1	6.298	0.720	-1.230	1.00	2.44	C
HETATM	74	C	MOL	A	1	6.715	0.892	3.378	1.00	15.70	C
HETATM	75	C	MOL	A	1	6.779	1.958	-7.419	1.00	10.67	C
HETATM	76	C	MOL	A	1	7.241	4.116	-1.155	1.00	1.99	C
HETATM	77	C	MOL	A	1	7.323	-3.342	5.671	1.00	4.64	C
HETATM	78	C	MOL	A	1	7.713	0.169	2.895	1.00	6.49	C
HETATM	79	C	MOL	A	1	7.716	0.305	0.273	1.00	-0.11	C
HETATM	80	C	MOL	A	1	8.626	1.225	3.961	1.00	8.55	C
HETATM	81	C	MOL	A	1	8.600	3.585	0.436	1.00	-1.29	C
HETATM	82	C	MOL	A	1	9.545	3.565	-0.439	1.00	-3.46	C
HETATM	83	C	MOL	A	1	10.489	1.198	-3.480	1.00	38.25	C
HETATM	84	C	MOL	A	1	12.318	0.643	-0.840	1.00	37.80	C
HETATM	1	O	MOL	A	1	-8.923	-0.847	0.370	1.00	-21.56	O
HETATM	2	O	MOL	A	1	-8.465	-3.079	3.779	1.00	-47.03	O
HETATM	3	O	MOL	A	1	-8.468	2.171	-2.583	1.00	-30.48	O
HETATM	4	O	MOL	A	1	-8.099	2.388	1.540	1.00	15.89	O
HETATM	5	O	MOL	A	1	-7.725	0.909	-9.236	1.00	20.83	O
HETATM	6	O	MOL	A	1	-7.351	1.554	-5.403	1.00	6.51	O
HETATM	7	O	MOL	A	1	-6.006	5.801	4.246	1.00	12.74	O
HETATM	8	O	MOL	A	1	-5.911	2.558	-3.455	1.00	9.68	O
HETATM	9	O	MOL	A	1	-5.840	-1.176	-5.433	1.00	-11.50	O
HETATM	10	O	MOL	A	1	-5.381	-4.150	1.594	1.00	-17.86	O
HETATM	11	O	MOL	A	1	-5.387	0.482	-0.865	1.00	12.58	O
HETATM	12	O	MOL	A	1	-5.192	4.242	0.529	1.00	-11.25	O
HETATM	13	O	MOL	A	1	-5.167	-3.597	-5.849	1.00	10.08	O
HETATM	14	O	MOL	A	1	-4.750	-1.925	-2.822	1.00	-1.86	O
HETATM	15	O	MOL	A	1	-4.482	-0.927	-6.622	1.00	-8.68	O
HETATM	16	O	MOL	A	1	-4.245	-3.783	-0.982	1.00	-18.04	O

HETATM	17	O	MOL	A	1	-4.319	1.688	-7.742	1.00	10.39	O
HETATM	18	O	MOL	A	1	-3.930	-3.233	6.635	1.00	-43.69	O
HETATM	19	O	MOL	A	1	-3.754	-1.003	-4.458	1.00	5.88	O
HETATM	20	O	MOL	A	1	-3.773	0.495	-0.588	1.00	-1.61	O
HETATM	21	O	MOL	A	1	-3.286	0.668	-4.469	1.00	-0.94	O
HETATM	22	O	MOL	A	1	-3.242	1.591	2.739	1.00	-9.60	O
HETATM	23	O	MOL	A	1	-3.145	-0.622	-3.453	1.00	1.94	O
HETATM	24	O	MOL	A	1	-2.747	5.491	-3.572	1.00	-2.61	O
HETATM	25	O	MOL	A	1	-2.667	1.484	-4.123	1.00	-1.08	O
HETATM	26	O	MOL	A	1	-2.509	-0.939	2.466	1.00	-19.39	O
HETATM	27	O	MOL	A	1	-2.509	4.008	1.074	1.00	-18.07	O
HETATM	28	O	MOL	A	1	-1.794	7.634	-1.662	1.00	-3.40	O
HETATM	29	O	MOL	A	1	-1.320	-4.129	-7.350	1.00	15.71	O
HETATM	30	O	MOL	A	1	-1.334	5.693	4.928	1.00	-14.67	O
HETATM	31	O	MOL	A	1	-1.051	1.935	-6.914	1.00	-13.51	O
HETATM	32	O	MOL	A	1	-1.038	3.348	-3.567	1.00	-15.57	O
HETATM	33	O	MOL	A	1	-0.858	-8.435	2.820	1.00	-44.82	O
HETATM	34	O	MOL	A	1	-0.737	3.618	6.405	1.00	-41.85	O
HETATM	35	O	MOL	A	1	-0.524	-5.019	3.600	1.00	-10.39	O
HETATM	36	O	MOL	A	1	-0.445	-3.120	1.471	1.00	-40.23	O
HETATM	37	O	MOL	A	1	-0.297	-1.578	5.991	1.00	-19.33	O
HETATM	38	O	MOL	A	1	-0.195	3.366	0.923	1.00	-5.22	O
HETATM	39	O	MOL	A	1	-0.182	7.564	1.645	1.00	-26.18	O
HETATM	40	O	MOL	A	1	0.111	-1.268	-1.811	1.00	-18.19	O
HETATM	41	O	MOL	A	1	0.411	-4.305	1.359	1.00	-20.66	O
HETATM	42	O	MOL	A	1	0.507	0.413	6.630	1.00	-11.11	O
HETATM	43	O	MOL	A	1	0.511	3.039	-0.271	1.00	-7.32	O
HETATM	44	O	MOL	A	1	0.690	-7.338	-1.062	1.00	-7.71	O
HETATM	45	O	MOL	A	1	0.726	-4.940	-4.093	1.00	-9.44	O
HETATM	46	O	MOL	A	1	0.753	2.632	1.393	1.00	-0.58	O
HETATM	47	O	MOL	A	1	0.865	4.697	-2.802	1.00	-17.65	O
HETATM	48	O	MOL	A	1	0.993	0.656	-3.052	1.00	-2.23	O
HETATM	49	O	MOL	A	1	1.123	-3.270	2.575	1.00	-36.47	O
HETATM	50	O	MOL	A	1	1.205	-5.022	-2.216	1.00	-11.86	O
HETATM	51	O	MOL	A	1	1.418	3.725	0.677	1.00	0.12	O
HETATM	52	O	MOL	A	1	1.468	1.764	2.111	1.00	-11.40	O
HETATM	53	O	MOL	A	1	1.611	4.235	-7.107	1.00	-13.96	O
HETATM	54	O	MOL	A	1	1.671	-1.177	6.285	1.00	-20.78	O
HETATM	55	O	MOL	A	1	1.802	-3.528	2.125	1.00	-22.62	O
HETATM	56	O	MOL	A	1	2.254	-3.196	1.437	1.00	-24.70	O
HETATM	57	O	MOL	A	1	2.254	-2.716	2.408	1.00	-36.58	O
HETATM	58	O	MOL	A	1	2.277	3.439	2.424	1.00	-15.60	O
HETATM	59	O	MOL	A	1	2.553	4.849	6.596	1.00	-51.43	O
HETATM	60	O	MOL	A	1	2.630	-2.218	-1.706	1.00	-8.33	O
HETATM	61	O	MOL	A	1	2.984	-2.196	2.117	1.00	-34.87	O
HETATM	62	O	MOL	A	1	3.524	-5.638	3.814	1.00	-4.81	O
HETATM	63	O	MOL	A	1	3.518	0.567	1.627	1.00	-29.06	O
HETATM	64	O	MOL	A	1	3.509	5.855	0.803	1.00	-31.12	O
HETATM	65	O	MOL	A	1	3.650	1.125	-6.149	1.00	-9.66	O
HETATM	66	O	MOL	A	1	4.488	-4.991	-0.683	1.00	-31.32	O
HETATM	67	O	MOL	A	1	4.697	1.511	7.251	1.00	-51.37	O

HETATM	68	O	MOL	A	1	5.198	0.580	-0.503	1.00	-9.90	O
HETATM	69	O	MOL	A	1	5.826	-3.672	0.563	1.00	-28.95	O
HETATM	70	O	MOL	A	1	6.151	3.025	2.659	1.00	-30.75	O
HETATM	71	O	MOL	A	1	6.653	3.998	-6.910	1.00	3.56	O
HETATM	72	O	MOL	A	1	6.841	4.341	-0.473	1.00	0.12	O
HETATM	73	O	MOL	A	1	8.211	2.237	-4.078	1.00	-27.72	O
HETATM	74	O	MOL	A	1	9.715	-0.383	-1.039	1.00	-4.35	O
HETATM	75	O	MOL	A	1	10.843	3.292	-1.134	1.00	-12.78	O
HETATM	76	O	MOL	A	1	11.025	1.276	1.852	1.00	-32.23	O

Table S2. The electrostatic potential (ESP) of Model-2 with 5-coordinated (at B3LYP/6-311++G (d, p) level)). Unit of B-factor field (i.e. ESP) is kcal/mol.

HETATM	1	C	MOL	A	1	-9.141	0.344	-6.999	1.00	19.76	C
HETATM	2	C	MOL	A	1	-7.692	3.519	-6.112	1.00	46.92	C
HETATM	3	C	MOL	A	1	-7.622	-0.129	-8.977	1.00	14.59	C
HETATM	4	C	MOL	A	1	-7.295	2.348	-9.457	1.00	19.61	C
HETATM	5	C	MOL	A	1	-7.227	1.972	0.707	1.00	23.18	C
HETATM	6	C	MOL	A	1	-6.174	0.602	5.894	1.00	60.45	C
HETATM	7	C	MOL	A	1	-5.957	-1.123	-8.382	1.00	13.52	C
HETATM	8	C	MOL	A	1	-5.622	4.822	0.786	1.00	27.32	C
HETATM	9	C	MOL	A	1	-5.280	-2.548	-6.451	1.00	7.14	C
HETATM	10	C	MOL	A	1	-5.318	6.141	-1.119	1.00	59.70	C
HETATM	11	C	MOL	A	1	-5.171	0.291	-0.241	1.00	32.54	C
HETATM	12	C	MOL	A	1	-4.884	-1.359	0.510	1.00	18.20	C
HETATM	13	C	MOL	A	1	-4.528	-5.528	5.196	1.00	-3.11	C
HETATM	14	C	MOL	A	1	-4.282	-2.977	-0.395	1.00	-0.74	C
HETATM	15	C	MOL	A	1	-4.042	-1.229	-3.416	1.00	-3.90	C
HETATM	16	C	MOL	A	1	-3.907	0.660	2.999	1.00	12.02	C
HETATM	17	C	MOL	A	1	-3.857	-0.844	3.564	1.00	11.01	C
HETATM	18	C	MOL	A	1	-3.100	-1.055	-4.663	1.00	4.40	C
HETATM	19	C	MOL	A	1	-2.704	6.380	-2.783	1.00	8.11	C
HETATM	20	C	MOL	A	1	-2.319	-3.466	-8.582	1.00	19.26	C
HETATM	21	C	MOL	A	1	-2.004	-7.384	-2.658	1.00	51.49	C
HETATM	22	C	MOL	A	1	-2.056	0.675	-5.166	1.00	0.58	C
HETATM	23	C	MOL	A	1	-1.988	4.570	-3.148	1.00	2.21	C
HETATM	24	C	MOL	A	1	-1.923	0.655	3.142	1.00	7.99	C
HETATM	25	C	MOL	A	1	-1.415	-1.549	-1.521	1.00	-3.29	C
HETATM	26	C	MOL	A	1	-1.257	0.362	6.923	1.00	7.26	C
HETATM	27	C	MOL	A	1	-1.136	-4.660	1.702	1.00	3.99	C
HETATM	28	C	MOL	A	1	-1.102	-2.114	-3.394	1.00	12.08	C
HETATM	29	C	MOL	A	1	-1.129	6.748	-0.571	1.00	10.65	C
HETATM	30	C	MOL	A	1	-1.053	-3.150	5.937	1.00	-0.11	C
HETATM	31	C	MOL	A	1	-1.005	4.567	4.865	1.00	15.71	C
HETATM	32	C	MOL	A	1	-0.796	-4.097	0.312	1.00	1.82	C
HETATM	33	C	MOL	A	1	-0.771	-4.876	5.734	1.00	3.99	C
HETATM	34	C	MOL	A	1	-0.718	-4.763	-5.580	1.00	17.59	C
HETATM	35	C	MOL	A	1	-0.441	-2.511	-2.509	1.00	7.10	C
HETATM	36	C	MOL	A	1	-0.421	6.289	3.468	1.00	-3.34	C
HETATM	37	C	MOL	A	1	-0.199	3.644	-4.489	1.00	2.22	C
HETATM	38	C	MOL	A	1	-0.240	3.848	-0.084	1.00	5.10	C
HETATM	39	C	MOL	A	1	-0.238	4.400	-2.625	1.00	0.92	C
HETATM	40	C	MOL	A	1	0.014	-1.153	-8.031	1.00	23.04	C
HETATM	41	C	MOL	A	1	0.143	-0.957	5.992	1.00	6.31	C
HETATM	42	C	MOL	A	1	0.162	2.814	0.553	1.00	1.34	C
HETATM	43	C	MOL	A	1	0.207	5.262	-0.183	1.00	20.11	C
HETATM	44	C	MOL	A	1	0.438	-1.876	-5.961	1.00	29.05	C
HETATM	45	C	MOL	A	1	0.872	1.463	7.227	1.00	0.03	C
HETATM	46	C	MOL	A	1	1.028	-2.987	0.945	1.00	-0.38	C
HETATM	47	C	MOL	A	1	1.353	0.337	6.433	1.00	2.89	C
HETATM	48	C	MOL	A	1	1.686	1.795	1.909	1.00	3.18	C
HETATM	49	C	MOL	A	1	1.696	6.095	2.392	1.00	0.94	C

HETATM	50	C	MOL	A	1	1.808	-4.739	3.542	1.00	-1.47	C
HETATM	51	C	MOL	A	1	1.901	3.540	1.141	1.00	12.87	C
HETATM	52	C	MOL	A	1	1.901	5.476	-2.310	1.00	23.78	C
HETATM	53	C	MOL	A	1	2.219	4.265	1.026	1.00	13.37	C
HETATM	54	C	MOL	A	1	2.249	-8.226	1.927	1.00	3.63	C
HETATM	55	C	MOL	A	1	2.457	3.124	3.127	1.00	-4.88	C
HETATM	56	C	MOL	A	1	2.552	-7.245	-1.487	1.00	9.29	C
HETATM	57	C	MOL	A	1	2.588	-3.543	-4.850	1.00	48.98	C
HETATM	58	C	MOL	A	1	2.678	-2.595	-0.813	1.00	1.29	C
HETATM	59	C	MOL	A	1	2.774	-0.295	2.532	1.00	-8.31	C
HETATM	60	C	MOL	A	1	2.766	1.451	-10.092	1.00	15.00	C
HETATM	61	C	MOL	A	1	2.898	2.613	5.910	1.00	15.56	C
HETATM	62	C	MOL	A	1	3.022	-7.814	3.636	1.00	3.99	C
HETATM	63	C	MOL	A	1	3.255	-2.989	5.708	1.00	24.10	C
HETATM	64	C	MOL	A	1	3.324	0.520	-2.147	1.00	19.98	C
HETATM	65	C	MOL	A	1	3.476	0.497	2.506	1.00	-5.97	C
HETATM	66	C	MOL	A	1	4.228	2.827	4.690	1.00	-7.21	C
HETATM	67	C	MOL	A	1	4.297	4.090	-10.148	1.00	10.26	C
HETATM	68	C	MOL	A	1	4.852	-7.360	0.464	1.00	4.36	C
HETATM	69	C	MOL	A	1	4.938	4.726	-5.473	1.00	8.10	C
HETATM	70	C	MOL	A	1	5.382	4.853	0.862	1.00	3.03	C
HETATM	71	C	MOL	A	1	5.798	3.209	-2.855	1.00	17.56	C
HETATM	72	C	MOL	A	1	5.732	5.205	-6.426	1.00	9.14	C
HETATM	73	C	MOL	A	1	5.870	0.822	1.183	1.00	-1.22	C
HETATM	74	C	MOL	A	1	6.022	4.528	2.839	1.00	-2.17	C
HETATM	75	C	MOL	A	1	6.044	-4.976	3.884	1.00	9.33	C
HETATM	76	C	MOL	A	1	6.156	1.537	-8.025	1.00	9.91	C
HETATM	77	C	MOL	A	1	6.385	1.893	5.826	1.00	-5.52	C
HETATM	78	C	MOL	A	1	6.872	0.577	2.909	1.00	3.13	C
HETATM	79	C	MOL	A	1	6.909	1.711	-0.000	1.00	0.35	C
HETATM	80	C	MOL	A	1	7.140	1.257	2.253	1.00	1.57	C
HETATM	81	C	MOL	A	1	7.537	-3.265	5.549	1.00	5.36	C
HETATM	82	C	MOL	A	1	8.540	0.017	3.802	1.00	-0.72	C
HETATM	83	C	MOL	A	1	10.385	5.079	0.865	1.00	34.13	C
HETATM	84	C	MOL	A	1	10.649	4.400	4.045	1.00	35.05	C
HETATM	1	O	MOL	A	1	-8.477	0.912	-8.798	1.00	13.72	O
HETATM	2	O	MOL	A	1	-8.120	1.533	2.203	1.00	18.69	O
HETATM	3	O	MOL	A	1	-7.856	0.589	3.727	1.00	17.11	O
HETATM	4	O	MOL	A	1	-7.202	-0.992	1.128	1.00	-9.27	O
HETATM	5	O	MOL	A	1	-6.869	0.426	-5.127	1.00	-1.99	O
HETATM	6	O	MOL	A	1	-6.623	-4.447	2.913	1.00	35.94	O
HETATM	7	O	MOL	A	1	-5.469	-2.182	-1.743	1.00	26.35	O
HETATM	8	O	MOL	A	1	-5.281	-3.502	-6.078	1.00	5.55	O
HETATM	9	O	MOL	A	1	-5.159	1.466	-2.143	1.00	22.58	O
HETATM	10	O	MOL	A	1	-4.084	-5.544	-0.960	1.00	6.94	O
HETATM	11	O	MOL	A	1	-4.056	-3.475	6.908	1.00	37.97	O
HETATM	12	O	MOL	A	1	-3.673	0.150	-4.373	1.00	32.80	O
HETATM	13	O	MOL	A	1	-3.558	2.901	-0.006	1.00	28.83	O
HETATM	14	O	MOL	A	1	-3.100	-0.844	2.651	1.00	10.94	O
HETATM	15	O	MOL	A	1	-2.980	0.783	-4.730	1.00	25.84	O
HETATM	16	O	MOL	A	1	-2.930	5.593	-3.582	1.00	-2.41	O

HETATM	17	O	MOL	A	1	-2.837	1.239	-6.898	1.00	22.91	O
HETATM	18	O	MOL	A	1	-2.623	2.189	3.198	1.00	14.49	O
HETATM	19	O	MOL	A	1	-2.384	7.891	-1.751	1.00	11.16	O
HETATM	20	O	MOL	A	1	-1.706	1.758	-6.631	1.00	20.03	O
HETATM	21	O	MOL	A	1	-1.503	3.835	-3.959	1.00	19.00	O
HETATM	22	O	MOL	A	1	-1.107	-8.129	3.161	1.00	42.62	O
HETATM	23	O	MOL	A	1	-1.187	5.271	4.756	1.00	18.05	O
HETATM	24	O	MOL	A	1	-1.118	-4.417	-7.235	1.00	12.01	O
HETATM	25	O	MOL	A	1	-1.030	3.768	6.269	1.00	39.39	O
HETATM	26	O	MOL	A	1	-0.966	-1.158	-1.918	1.00	17.86	O
HETATM	27	O	MOL	A	1	-0.705	-3.437	1.342	1.00	15.93	O
HETATM	28	O	MOL	A	1	-0.718	5.903	-2.596	1.00	10.17	O
HETATM	29	O	MOL	A	1	-0.605	-1.876	6.059	1.00	10.27	O
HETATM	30	O	MOL	A	1	-0.635	3.126	0.075	1.00	11.90	O
HETATM	31	O	MOL	A	1	-0.508	7.486	1.642	1.00	28.49	O
HETATM	32	O	MOL	A	1	-0.245	2.863	-0.255	1.00	10.82	O
HETATM	33	O	MOL	A	1	0.152	0.671	6.897	1.00	-3.91	O
HETATM	34	O	MOL	A	1	0.218	-4.281	1.255	1.00	-7.08	O
HETATM	35	O	MOL	A	1	0.165	0.757	-3.091	1.00	0.26	O
HETATM	36	O	MOL	A	1	0.453	-7.708	-0.838	1.00	-3.06	O
HETATM	37	O	MOL	A	1	0.552	4.817	-2.310	1.00	-8.24	O
HETATM	38	O	MOL	A	1	0.853	-3.256	3.238	1.00	26.63	O
HETATM	39	O	MOL	A	1	1.028	-4.852	-2.427	1.00	-4.87	O
HETATM	40	O	MOL	A	1	1.120	4.736	-0.618	1.00	-8.54	O
HETATM	41	O	MOL	A	1	1.336	-3.477	2.292	1.00	15.00	O
HETATM	42	O	MOL	A	1	1.456	4.664	-6.723	1.00	17.67	O
HETATM	43	O	MOL	A	1	1.543	-1.123	-3.360	1.00	-2.38	O
HETATM	44	O	MOL	A	1	2.040	5.102	6.306	1.00	50.10	O
HETATM	45	O	MOL	A	1	2.167	2.979	2.612	1.00	-6.31	O
HETATM	46	O	MOL	A	1	2.467	-2.169	1.818	1.00	25.14	O
HETATM	47	O	MOL	A	1	2.533	-2.591	-1.748	1.00	-2.15	O
HETATM	48	O	MOL	A	1	2.768	-2.266	0.327	1.00	12.39	O
HETATM	49	O	MOL	A	1	2.749	-0.374	2.494	1.00	-9.80	O
HETATM	50	O	MOL	A	1	3.349	-5.651	4.076	1.00	-3.96	O
HETATM	51	O	MOL	A	1	3.409	1.293	-5.955	1.00	12.26	O
HETATM	52	O	MOL	A	1	3.356	1.954	2.232	1.00	23.35	O
HETATM	53	O	MOL	A	1	3.480	1.297	2.217	1.00	22.05	O
HETATM	54	O	MOL	A	1	3.965	3.567	3.313	1.00	22.21	O
HETATM	55	O	MOL	A	1	4.212	-4.811	-0.803	1.00	29.32	O
HETATM	56	O	MOL	A	1	4.464	1.239	7.305	1.00	46.67	O
HETATM	57	O	MOL	A	1	4.563	5.707	-2.230	1.00	26.06	O
HETATM	58	O	MOL	A	1	4.900	-2.838	5.040	1.00	-8.95	O
HETATM	59	O	MOL	A	1	4.989	-7.263	2.684	1.00	-3.79	O
HETATM	60	O	MOL	A	1	5.046	0.505	-0.104	1.00	15.22	O
HETATM	61	O	MOL	A	1	5.632	-3.557	0.353	1.00	31.06	O
HETATM	62	O	MOL	A	1	8.240	3.044	5.463	1.00	35.42	O
HETATM	63	O	MOL	A	1	8.331	5.635	2.996	1.00	-6.50	O
HETATM	64	O	MOL	A	1	8.781	4.622	-1.198	1.00	31.27	O
HETATM	65	O	MOL	A	1	10.064	2.155	2.053	1.00	15.35	O

Table S3. The electrostatic potential (ESP) of Model-3 with 4-coordinated (at B3LYP/6-311++G (d, p) level)). Unit of B-factor field (i.e. ESP) is kcal/mol.

HETATM	1	C	MOL	A	1	-7.411	1.432	2.758	1.00	60.48	C
HETATM	2	C	MOL	A	1	-5.907	1.828	1.249	1.00	58.83	C
HETATM	3	C	MOL	A	1	-5.894	2.352	-4.812	1.00	84.51	C
HETATM	4	C	MOL	A	1	-5.810	2.235	4.348	1.00	62.32	C
HETATM	5	C	MOL	A	1	-5.782	3.754	-9.031	1.00	49.59	C
HETATM	6	C	MOL	A	1	-5.735	4.330	3.700	1.00	59.05	C
HETATM	7	C	MOL	A	1	-5.346	-0.405	5.272	1.00	90.11	C
HETATM	8	C	MOL	A	1	-5.234	-1.560	-7.462	1.00	45.65	C
HETATM	9	C	MOL	A	1	-4.641	3.578	0.425	1.00	63.32	C
HETATM	10	C	MOL	A	1	-4.561	-3.518	2.598	1.00	59.18	C
HETATM	11	C	MOL	A	1	-4.345	1.435	0.423	1.00	59.84	C
HETATM	12	C	MOL	A	1	-4.169	-3.041	-4.783	1.00	45.83	C
HETATM	13	C	MOL	A	1	-3.998	-0.313	0.060	1.00	60.43	C
HETATM	14	C	MOL	A	1	-3.873	5.995	-0.851	1.00	98.24	C
HETATM	15	C	MOL	A	1	-3.771	-6.586	2.195	1.00	44.82	C
HETATM	16	C	MOL	A	1	-3.759	-2.377	-0.493	1.00	42.75	C
HETATM	17	C	MOL	A	1	-3.743	-2.120	-5.880	1.00	45.52	C
HETATM	18	C	MOL	A	1	-3.081	-6.091	0.417	1.00	44.98	C
HETATM	19	C	MOL	A	1	-2.992	5.606	1.335	1.00	63.01	C
HETATM	20	C	MOL	A	1	-2.860	-3.553	-1.433	1.00	80.05	C
HETATM	21	C	MOL	A	1	-2.281	1.832	-5.688	1.00	34.52	C
HETATM	22	C	MOL	A	1	-2.132	1.293	-4.905	1.00	35.15	C
HETATM	23	C	MOL	A	1	-1.449	2.864	-1.285	1.00	45.90	C
HETATM	24	C	MOL	A	1	-0.953	6.291	-5.320	1.00	47.14	C
HETATM	25	C	MOL	A	1	-0.829	-4.207	-4.636	1.00	89.45	C
HETATM	26	C	MOL	A	1	-0.829	2.937	4.328	1.00	53.31	C
HETATM	27	C	MOL	A	1	-0.352	-6.582	4.873	1.00	51.45	C
HETATM	28	C	MOL	A	1	-0.346	-4.521	4.904	1.00	49.12	C
HETATM	29	C	MOL	A	1	-0.400	0.812	2.455	1.00	55.95	C
HETATM	30	C	MOL	A	1	-0.099	-4.237	-2.193	1.00	68.77	C
HETATM	31	C	MOL	A	1	-0.113	-1.740	5.432	1.00	52.18	C
HETATM	32	C	MOL	A	1	-0.099	5.278	10.777	1.00	35.67	C
HETATM	33	C	MOL	A	1	0.184	-6.900	2.907	1.00	48.15	C
HETATM	34	C	MOL	A	1	0.216	-4.041	1.176	1.00	50.27	C
HETATM	35	C	MOL	A	1	0.304	8.670	-2.308	1.00	55.09	C
HETATM	36	C	MOL	A	1	0.325	2.877	4.141	1.00	54.85	C
HETATM	37	C	MOL	A	1	0.786	1.605	0.563	1.00	64.49	C
HETATM	38	C	MOL	A	1	0.729	3.771	-3.745	1.00	42.65	C
HETATM	39	C	MOL	A	1	0.933	7.052	-3.225	1.00	57.16	C
HETATM	40	C	MOL	A	1	1.250	8.102	2.605	1.00	46.53	C
HETATM	41	C	MOL	A	1	1.284	-3.867	3.274	1.00	57.49	C
HETATM	42	C	MOL	A	1	1.330	-0.708	5.833	1.00	46.35	C
HETATM	43	C	MOL	A	1	1.383	5.160	-1.617	1.00	69.26	C
HETATM	44	C	MOL	A	1	1.513	-4.421	3.173	1.00	57.22	C
HETATM	45	C	MOL	A	1	2.055	0.491	6.588	1.00	44.87	C
HETATM	46	C	MOL	A	1	2.351	5.148	2.283	1.00	53.13	C
HETATM	47	C	MOL	A	1	2.425	-0.903	-4.019	1.00	57.67	C
HETATM	48	C	MOL	A	1	2.500	-3.254	-4.552	1.00	46.86	C
HETATM	49	C	MOL	A	1	2.585	-0.079	1.627	1.00	60.63	C

HETATM	50	C	MOL	A	1	2.637	2.980	-0.649	1.00	54.40	C
HETATM	51	C	MOL	A	1	2.758	-3.210	-0.112	1.00	43.20	C
HETATM	52	C	MOL	A	1	2.854	-2.672	-1.747	1.00	38.01	C
HETATM	53	C	MOL	A	1	3.314	4.621	-3.902	1.00	67.32	C
HETATM	54	C	MOL	A	1	3.486	-4.968	4.590	1.00	77.25	C
HETATM	55	C	MOL	A	1	3.719	0.724	5.702	1.00	40.67	C
HETATM	56	C	MOL	A	1	4.180	-0.181	-4.234	1.00	58.32	C
HETATM	57	C	MOL	A	1	4.239	-2.228	-0.974	1.00	35.06	C
HETATM	58	C	MOL	A	1	4.252	-1.769	1.942	1.00	59.70	C
HETATM	59	C	MOL	A	1	5.155	-2.510	1.823	1.00	55.73	C
HETATM	60	C	MOL	A	1	5.279	-0.987	-5.650	1.00	47.86	C
HETATM	61	C	MOL	A	1	5.803	-4.252	4.127	1.00	54.85	C
HETATM	62	C	MOL	A	1	6.188	-5.200	-4.506	1.00	45.68	C
HETATM	63	C	MOL	A	1	6.410	-3.848	-1.681	1.00	52.44	C
HETATM	64	C	MOL	A	1	6.412	0.643	3.162	1.00	01.45	C
HETATM	65	C	MOL	A	1	6.463	0.945	-3.904	1.00	54.25	C
HETATM	66	C	MOL	A	1	7.227	1.371	-1.275	1.00	55.27	C
HETATM	67	C	MOL	A	1	7.423	-5.320	-5.190	1.00	45.47	C
HETATM	68	C	MOL	A	1	7.509	-1.125	-4.863	1.00	46.27	C
HETATM	69	C	MOL	A	1	7.878	-3.725	-1.483	1.00	54.78	C
HETATM	70	C	MOL	A	1	7.921	-0.867	0.226	1.00	58.33	C
HETATM	71	C	MOL	A	1	8.235	-4.976	0.392	1.00	67.46	C
HETATM	72	C	MOL	A	1	8.474	-4.320	1.573	1.00	67.17	C
HETATM	73	C	MOL	A	1	9.592	-2.765	4.356	1.00	54.71	C
HETATM	1	O	MOL	A	1	-6.885	1.355	4.034	1.00	59.08	O
HETATM	2	O	MOL	A	1	-6.468	-2.540	3.070	1.00	30.23	O
HETATM	3	O	MOL	A	1	-6.151	-2.227	-4.225	1.00	37.32	O
HETATM	4	O	MOL	A	1	-6.125	4.218	-7.633	1.00	46.60	O
HETATM	5	O	MOL	A	1	-5.930	3.737	1.828	1.00	54.31	O
HETATM	6	O	MOL	A	1	-5.726	-0.148	0.704	1.00	40.87	O
HETATM	7	O	MOL	A	1	-5.696	0.680	-7.358	1.00	42.38	O
HETATM	8	O	MOL	A	1	-5.223	-2.737	0.418	1.00	28.47	O
HETATM	9	O	MOL	A	1	-3.919	0.796	-1.587	1.00	14.50	O
HETATM	10	O	MOL	A	1	-3.890	-4.346	0.076	1.00	22.83	O
HETATM	11	O	MOL	A	1	-3.511	-5.125	4.669	1.00	9.11	O
HETATM	12	O	MOL	A	1	-3.419	-3.318	-3.101	1.00	24.32	O
HETATM	13	O	MOL	A	1	-3.258	-1.094	-1.911	1.00	17.73	O
HETATM	14	O	MOL	A	1	-2.871	2.302	-4.605	1.00	24.63	O
HETATM	15	O	MOL	A	1	-2.366	6.912	-3.154	1.00	52.50	O
HETATM	16	O	MOL	A	1	-2.079	0.650	-9.055	1.00	-0.76	O
HETATM	17	O	MOL	A	1	-1.845	5.448	0.700	1.00	45.72	O
HETATM	18	O	MOL	A	1	-1.386	2.350	-0.332	1.00	24.57	O
HETATM	19	O	MOL	A	1	-0.885	7.004	-9.926	1.00	27.90	O
HETATM	20	O	MOL	A	1	-0.819	0.878	2.365	1.00	43.51	O
HETATM	21	O	MOL	A	1	-0.747	8.341	-3.293	1.00	51.74	O
HETATM	22	O	MOL	A	1	-0.655	-0.251	-5.447	1.00	7.20	O
HETATM	23	O	MOL	A	1	-0.670	2.835	-0.496	1.00	28.02	O
HETATM	24	O	MOL	A	1	-0.490	1.443	6.339	1.00	6.55	O
HETATM	25	O	MOL	A	1	-0.413	2.312	-0.804	1.00	27.60	O
HETATM	26	O	MOL	A	1	-0.398	9.554	2.138	1.00	38.13	O
HETATM	27	O	MOL	A	1	-0.281	1.586	-5.527	1.00	7.20	O

HETATM	28	O	MOL	A	1	-0.282	4.728	3.631	1.00	34.46	O
HETATM	29	O	MOL	A	1	-0.013	-6.811	-1.121	1.00	-0.52	O
HETATM	30	O	MOL	A	1	0.060	5.007	-4.177	1.00	31.39	O
HETATM	31	O	MOL	A	1	0.304	5.890	1.577	1.00	34.82	O
HETATM	32	O	MOL	A	1	0.328	-3.153	5.017	1.00	35.01	O
HETATM	33	O	MOL	A	1	0.695	-6.457	3.478	1.00	47.37	O
HETATM	34	O	MOL	A	1	0.640	4.689	-8.970	1.00	28.29	O
HETATM	35	O	MOL	A	1	0.680	6.034	-8.993	1.00	28.45	O
HETATM	36	O	MOL	A	1	1.112	-4.573	-0.713	1.00	8.47	O
HETATM	37	O	MOL	A	1	1.338	-2.064	5.084	1.00	39.17	O
HETATM	38	O	MOL	A	1	1.844	3.181	-5.057	1.00	23.40	O
HETATM	39	O	MOL	A	1	1.837	4.959	-0.076	1.00	32.81	O
HETATM	40	O	MOL	A	1	2.044	-4.089	1.050	1.00	24.17	O
HETATM	41	O	MOL	A	1	2.208	7.314	-0.088	1.00	40.81	O
HETATM	42	O	MOL	A	1	2.687	3.590	-1.993	1.00	28.22	O
HETATM	43	O	MOL	A	1	3.002	1.115	1.173	1.00	50.41	O
HETATM	44	O	MOL	A	1	3.357	0.324	0.584	1.00	41.52	O
HETATM	45	O	MOL	A	1	3.636	3.435	5.469	1.00	2.40	O
HETATM	46	O	MOL	A	1	3.707	-3.249	-2.230	1.00	25.54	O
HETATM	47	O	MOL	A	1	3.755	-3.900	-6.400	1.00	35.38	O
HETATM	48	O	MOL	A	1	3.870	-2.494	-0.272	1.00	27.34	O
HETATM	49	O	MOL	A	1	4.239	-1.343	-4.072	1.00	34.70	O
HETATM	50	O	MOL	A	1	4.407	-1.669	7.495	1.00	-2.39	O
HETATM	51	O	MOL	A	1	4.503	-3.090	-0.334	1.00	28.53	O
HETATM	52	O	MOL	A	1	5.060	-3.576	2.657	1.00	40.83	O
HETATM	53	O	MOL	A	1	5.126	-3.096	-6.942	1.00	34.88	O
HETATM	54	O	MOL	A	1	6.092	-1.105	-4.106	1.00	35.31	O
HETATM	55	O	MOL	A	1	6.054	4.206	-2.815	1.00	7.21	O
HETATM	56	O	MOL	A	1	6.962	-1.265	-2.875	1.00	34.01	O
HETATM	57	O	MOL	A	1	7.304	-1.640	-1.681	1.00	37.60	O
HETATM	58	O	MOL	A	1	7.948	-0.122	0.700	1.00	55.78	O
HETATM	59	O	MOL	A	1	8.604	-3.183	-5.332	1.00	35.15	O
HETATM	60	O	MOL	A	1	10.202	-2.698	3.150	1.00	52.65	O

Table S4. Cartesian coordinates of 6-coordinated.

Reactant 1

C	-4.56248300	2.67228600	-4.91226000
H	-4.32349400	2.33624300	-3.90434700
C	-3.28772200	3.21885600	-5.58182200
H	-2.51066500	2.45304300	-5.64644200
H	-3.48744600	3.58731400	-6.59363400
H	-2.88782200	4.04802500	-4.99228200
C	-5.14555700	1.49136300	-5.66931300
O	-4.88439900	0.31026500	-5.38598700
N	-5.94926600	1.82245500	-6.70988200
H	-6.11236400	2.80342800	-6.88053900
C	-6.58348400	0.85828900	-7.58824300
H	-6.21465900	0.95279400	-8.61545900
H	-6.34244600	-0.13671900	-7.21434900
C	6.84651800	-1.16671500	4.67274700
H	7.78383200	-0.64513800	4.44508700
H	7.10012900	-2.06990200	5.23353800
C	6.10316900	-1.54016900	3.43320500
N	5.77535500	-0.64383000	2.43066500
H	6.02688000	0.33840200	2.37079500
C	5.05999800	-1.31140500	1.48786300
H	4.66992300	-0.81991200	0.61280600
N	4.90064700	-2.58091200	1.81222900
C	5.55217300	-2.72933100	3.02244600
H	5.59135200	-3.67981500	3.53567100
C	-1.79348100	-3.57171700	4.08574600
H	-0.92702400	-3.47568700	3.42913000
C	-3.04785400	-4.00976400	3.29386600
H	-2.88824300	-5.01587200	2.88837200
H	-3.91087100	-4.04440100	3.96034500
C	-3.32544200	-3.07211300	2.11633700
O	-2.33826000	-2.80539500	1.38158600
O	-4.50067000	-2.61195900	1.91430700
C	-2.15138000	-2.24506800	4.77012500
O	-3.13210700	-2.16842400	5.51412700
N	-1.39717500	-1.15067800	4.44160700
H	-0.71389700	-1.25601000	3.69027200
C	-1.91884900	0.16941800	4.76916200
H	-2.58924600	0.53025800	3.98010600
H	-2.52592900	0.06193100	5.66994700
C	-0.94204600	1.31279400	5.01213300
O	-1.39704900	2.42364100	5.30706800
N	0.37301800	1.05915400	4.83056900
H	0.64660700	0.09383000	4.68977400
C	1.44871700	2.01104000	5.09982100
H	1.58314200	2.14560900	6.17850300
C	2.77222900	1.48441800	4.49094300
H	2.63564800	1.30835600	3.41850200
H	3.52570100	2.26161700	4.62871000

C	3.30547600	0.23533200	5.19679700
O	4.08529400	0.32331600	6.14154800
N	2.82119900	-0.95383900	4.74542900
H	3.22119000	-1.78153000	5.16415000
H	2.32970400	-1.07214700	3.85843500
C	1.22550600	3.43521600	4.55632200
O	1.72393600	4.38328200	5.15037500
N	0.58358500	3.55431300	3.35859000
H	0.11317900	2.76116400	2.93668600
C	0.42331700	4.84275800	2.72918700
H	0.94269100	5.57286000	3.35900000
H	0.90547500	4.86571600	1.74523800
C	-1.01716700	5.31336400	2.53421100
O	-1.24280700	6.16433900	1.64154400
N	-1.94821400	4.81334900	3.34997400
H	-1.66636400	4.07255900	3.99338100
C	-3.37748100	5.04628700	3.18374300
H	-3.50693300	5.67920000	2.30362900
C	-4.18688800	3.75297400	3.05329300
H	-3.99306000	3.31632200	2.07189400
H	-3.82280600	3.03338500	3.79486100
C	-5.68564100	4.01131000	3.26281700
H	-5.88564800	4.21827300	4.32272200
H	-5.99833500	4.91000300	2.71334300
C	-6.58905600	2.87176700	2.77179600
H	-7.63670800	3.08461400	3.00563900
H	-6.51828500	2.79428400	1.68608800
N	-6.27912900	1.56343700	3.36251900
H	-6.62409000	1.38884600	4.29567800
C	-5.48814500	0.61370900	2.83915800
N	-5.10248100	0.64923500	1.54070100
H	-5.24371700	1.50602700	0.98537600
H	-4.17275400	0.24632100	1.35327000
N	-5.07722500	-0.41243800	3.57928700
H	-5.36723000	-0.52004900	4.53950400
H	-4.81370000	-1.27730700	3.08662600
N	-2.93944900	6.49228300	-0.61425400
H	-3.86588200	6.83162400	-0.81757700
C	-2.56434100	5.29067100	-1.10052000
N	-1.34428100	4.84266500	-0.80402200
H	-0.88416900	5.33106900	-0.03281400
H	-1.14677000	3.85026100	-0.88827400
N	-3.36232700	4.61123200	-1.94250300
H	-4.35665300	4.69142300	-1.76697400
H	-3.08868700	3.63513700	-2.14785900
C	4.86951900	3.46828500	-7.77924000
H	4.82030100	3.51631000	-8.87168700
H	5.70978900	2.81061600	-7.52257800
C	3.57211500	2.96847500	-7.18805400
C	3.10743900	3.42961000	-5.95068700
H	3.67439800	4.19123700	-5.41971600

C	1.92837700	2.94868200	-5.37771400
H	1.59503700	3.32517700	-4.41498000
C	1.18166200	1.97702800	-6.05258800
O	0.02086000	1.45150600	-5.54922000
H	-0.18532900	1.76604300	-4.62609400
C	1.61952400	1.51297100	-7.29781600
H	1.02440400	0.77335700	-7.82532700
C	2.79851400	2.00557900	-7.85049900
H	3.12336600	1.63436900	-8.81967300
M	-2.00753600	-1.63325200	-0.29927000
O	-0.53791000	1.67610800	-3.02431000
P	-1.59526400	1.14939400	-2.04494500
O	-2.97667700	1.84307300	-2.11974800
O	-1.71809000	-0.39174500	-2.02089600
O	-0.92610600	1.65545400	-0.60127100
P	-1.38892600	1.23107400	0.91351600
O	-2.40526000	0.09584900	0.79291700
O	-1.70367700	2.45376200	1.70280800
O	0.01809200	0.63734900	1.50207400
P	0.74720800	-0.82742000	1.26177300
O	-0.00905400	-1.54180800	0.14993500
O	0.94873200	-1.45809400	2.60701400
O	2.18509500	-0.38844200	0.66783600
C	2.23485500	0.30061600	-0.59602600
H	1.27875500	0.23034000	-1.12024800
H	3.00296200	-0.19302700	-1.20035300
C	2.61054200	1.74597900	-0.36148800
H	1.91683200	2.19470200	0.35835200
O	3.94905700	1.77116300	0.16032000
C	4.59934600	2.98474100	-0.20110200
H	4.79323700	3.58122900	0.69434700
N	5.89879900	2.66619100	-0.78453400
C	6.28010300	2.58472500	-2.11565200
H	5.58857300	2.80632800	-2.91623500
N	7.52941000	2.23280200	-2.29366500
C	8.00836900	2.05028300	-1.00893100
C	9.27428000	1.67636400	-0.51013200
N	10.32824200	1.43568800	-1.32181400
H	11.17805100	1.07987300	-0.91495700
H	10.19548200	1.41208600	-2.31935700
N	9.44182600	1.57002000	0.82121900
C	8.38993900	1.81505900	1.61448000
H	8.57697500	1.70506000	2.68077700
N	7.14273500	2.16878100	1.27491700
C	7.01494200	2.29228300	-0.05654200
C	2.66847100	2.63549800	-1.61677100
H	3.11397300	2.07528600	-2.45436600
O	1.45716400	3.24939600	-1.98808100
H	0.78166600	2.59327800	-2.29082500
C	3.64179800	3.73848700	-1.14534500
H	4.16581000	4.22657000	-1.97724500

O	2.93836500	4.69494200	-0.37553300
H	2.08011300	4.79026000	-0.82209400
C	-2.04048600	-4.37771800	-3.32125000
H	-1.06042500	-4.12910800	-3.74448100
C	-3.10212800	-3.34447900	-3.73714000
H	-4.07024300	-3.64167100	-3.31823400
H	-2.87381500	-2.37998900	-3.27645300
C	-3.24406200	-3.20279200	-5.25803400
H	-3.55109900	-4.16773200	-5.68316000
H	-4.05947200	-2.49962200	-5.46846100
C	-1.99137800	-2.72833900	-6.01360000
H	-1.15137400	-3.41891200	-5.86970100
H	-2.21862800	-2.76047700	-7.08584000
C	-1.53068600	-1.29691800	-5.72147900
H	-0.93889100	-0.91880400	-6.55688200
H	-2.38478400	-0.62524600	-5.59668200
N	-0.65329500	-1.13641200	-4.50927200
H	-1.16828300	-1.01953900	-3.60486900
H	0.04616200	-1.90924100	-4.37789200
H	-0.14333400	-0.24954200	-4.63752000
C	-1.84332000	-4.42091700	-1.83711500
O	-1.54808800	-3.38368600	-1.23121100
N	-1.96157700	-5.60807500	-1.21673300
H	-2.14324800	-6.42255100	-1.78464500
C	-1.85624600	-5.78101500	0.22626100
H	-1.94892300	-4.78793300	0.67265400
H	-2.67110200	-6.41856900	0.57456100
C	-0.55399500	-6.41978300	0.72263700
O	-0.59717500	-7.32415400	1.55341000
N	0.58570500	-5.88972400	0.21411800
H	0.56033400	-5.08948800	-0.41538200
C	1.88651600	-6.39070900	0.61174100
H	1.69853200	-7.25408000	1.25296400
C	2.78391600	-5.39651600	1.35965400
H	2.19582300	-4.92601500	2.16200700
C	3.97884900	-6.14071100	1.96139700
H	4.53124900	-6.67088800	1.17809000
H	4.66853300	-5.44253600	2.44083800
H	3.65133800	-6.86754600	2.71202100
O	3.23658500	-4.39697700	0.43917800
H	3.82291200	-3.75323000	0.92525000
O	-4.05016400	-1.95294200	-0.73361000
H	-4.48517300	-1.27584100	-1.29393900
H	-4.44258900	-2.05258700	0.16733300
O	-5.24565100	2.72366300	-0.42525300
H	-4.44318000	2.36747100	-0.85277600
H	-6.00116600	2.30884600	-0.92361900
O	1.39286600	-3.47718400	-1.30159700
H	0.87656900	-2.81316200	-0.79504500
H	2.16446000	-3.72051300	-0.72922400
O	-4.89399300	-0.03290300	-2.58440800

H	-4.85197200	-0.13930400	-3.55830400
H	-4.16589000	0.61260900	-2.39328700
O	1.11041900	-3.18058500	-3.91111600
H	1.27001200	-3.28720100	-2.92699300
H	1.97032500	-3.29857200	-4.33132900
O	-7.14049900	1.32715200	-1.75311200
H	-6.44021200	0.82783000	-2.22819100
H	-7.45928100	0.66067200	-1.11507600
O	-7.30656600	-2.31627700	2.67370800
H	-7.57817300	-1.99787200	1.79945400
H	-6.45598600	-2.75035800	2.49826200
O	-7.19004400	-0.89358900	0.06250500
H	-6.45073100	-0.50866800	0.57551200
H	-6.77598600	-1.56125900	-0.50160400
H	-2.31273100	-5.36564300	-3.71510200
H	-7.67051000	0.98712500	-7.58955700
H	2.43925900	-6.73407700	-0.27372200
H	-3.74122200	5.62222000	4.04727900
H	-2.49566700	6.76979800	0.26336000
H	-1.57847800	-4.31109900	4.86300600
H	5.11770600	4.46834600	-7.41136500
H	6.24319600	-0.52505000	5.32511200
H	-5.31552000	3.46695900	-4.83943900

Transition State 1

C	45.21099600	45.24400300	45.24400300
H	45.54487500	44.87742400	44.87742400
C	46.39332500	45.90364000	45.90364000
H	47.22233200	45.20400500	45.20400500
H	46.10061000	46.28424800	46.28424800
H	46.76597000	46.74750100	46.74750100
C	44.63162100	44.06107800	44.06107800
O	44.88026600	42.87993700	42.87993700
N	43.83411000	44.39431100	44.39431100
H	43.67864800	45.37571400	45.37571400
C	43.18999900	43.43000300	43.43000300
H	43.54674500	43.52530000	43.52530000
H	43.43640900	42.43520200	42.43520200
C	56.61999800	41.40499800	41.40499800
H	57.58229600	41.80865400	41.80865400
H	56.81323900	40.58302900	40.58302900
C	55.80878000	40.91678400	40.91678400
N	55.79416800	41.56848700	41.56848700
H	56.34437000	42.38488200	42.38488200
C	54.92739700	40.96732300	40.96732300
H	54.68564100	41.30972600	41.30972600
N	54.37418800	39.94394100	39.94394100
C	54.90964300	39.88828500	39.88828500
H	54.60429500	39.13796800	39.13796800
C	47.98000100	38.99999800	38.99999800
H	48.70056300	39.10047100	39.10047100

C	46.59137900	38.61001200	38.61001200
H	46.59283100	37.53980700	37.53980700
H	45.80037600	38.77516600	38.77516600
C	46.30644000	39.35438100	39.35438100
O	47.28138200	39.44491300	39.44491300
O	45.14600400	39.84073500	39.84073500
C	47.85402800	40.28208300	40.28208300
O	47.54507500	40.24552300	40.24552300
N	48.03202200	41.47520100	41.47520100
H	48.15764600	41.44378800	41.44378800
C	47.57565900	42.72452000	42.72452000
H	46.72595000	43.12886700	43.12886700
H	47.22750500	42.48145400	42.48145400
C	48.57485600	43.87622200	43.87622200
O	48.14648100	45.01350700	45.01350700
N	49.87847500	43.55350600	43.55350600
H	50.08185300	42.57336500	42.57336500
C	51.01441900	44.44032300	44.44032300
H	51.19801800	44.54388700	44.54388700
C	52.28094100	43.84331200	43.84331200
H	52.04190200	43.60812800	43.60812800
H	53.06849600	44.59816000	44.59816000
C	52.81674200	42.61791400	42.61791400
O	53.70574400	42.71286500	42.71286500
N	52.19495500	41.44017100	41.44017100
H	52.54211000	40.63896500	40.63896500
H	51.72400100	41.23897400	41.23897400
C	50.86251900	45.89530000	45.89530000
O	51.33570400	46.80602100	46.80602100
N	50.32045600	46.06586200	46.06586200
H	49.85340200	45.28791400	45.28791400
C	50.19993400	47.38117400	47.38117400
H	50.69690300	48.08684100	48.08684100
H	50.70953400	47.44269200	47.44269200
C	48.76801300	47.87007500	47.87007500
O	48.55953800	48.77237400	48.77237400
N	47.81447600	47.31769800	47.31769800
H	48.07389400	46.55170600	46.55170600
C	46.39600100	47.61800000	47.61800000
H	46.30149000	48.49418100	48.49418100
C	45.61049500	46.42663300	46.42663300
H	45.91169500	46.24756700	46.24756700
H	45.90259200	45.54080200	45.54080200
C	44.09500700	46.64236800	46.64236800
H	43.79689200	46.76762800	46.76762800
H	43.81511200	47.57403700	47.57403700
C	43.26441600	45.51826400	45.51826400
H	42.19726400	45.74761200	45.74761200
H	43.49649500	45.43674900	45.43674900
N	43.45214600	44.20756700	44.20756700
H	42.94752900	44.04126500	44.04126500

C	44.40381300	43.29883200	43.29883200
N	45.17686200	43.40038000	43.40038000
H	45.13709700	44.24752300	44.24752300
H	46.08790200	42.89428400	42.89428400
N	44.53974700	42.24150300	42.24150300
H	43.67706000	41.88918000	41.88918000
H	45.03398900	41.43934400	41.43934400
N	46.83403300	49.06399700	49.06399700
H	45.85756700	49.21943200	49.21943200
C	47.41636800	47.94647000	47.94647000
N	48.69026400	47.69780300	47.69780300
H	49.05314800	48.20495000	48.20495000
H	49.04341600	46.75828700	46.75828700
N	46.75531400	47.15696500	47.15696500
H	45.77316700	46.98613500	46.98613500
H	47.19614700	46.25828500	46.25828500
C	54.64300100	46.03999900	46.03999900
H	54.50626100	46.43639100	46.43639100
H	55.35336500	45.20677500	45.20677500
C	53.32706100	45.59925800	45.59925800
C	53.01850700	45.84111900	45.84111900
H	53.72641500	46.39159100	46.39159100
C	51.81981300	45.41112100	45.41112100
H	51.60990100	45.61769900	45.61769900
C	50.88801100	44.71342800	44.71342800
O	49.69902000	44.25122200	44.25122200
H	49.62369700	44.34530400	44.34530400
C	51.17069600	44.47522700	44.47522700
H	50.43707000	43.94990600	43.94990600
C	52.37083200	44.91268200	44.91268200
H	52.56817100	44.71641400	44.71641400
M	47.83174400	40.55007600	40.55007600
O	49.48255100	43.78347000	43.78347000
P	48.40036800	43.38178600	43.38178600
O	47.07309000	44.16396400	44.16396400
O	48.18527300	41.83941900	41.83941900
O	49.06611700	43.85474600	43.85474600
P	48.94845100	43.18695000	43.18695000
O	47.73029700	42.21863400	42.21863400
O	48.73326000	44.37097000	44.37097000
O	50.28787700	42.41907300	42.41907300
P	50.96530400	40.50036800	40.50036800
O	49.84395600	40.09758000	40.09758000
O	51.04503000	40.33493500	40.33493500
O	52.24634500	41.13464700	41.13464700
C	52.06178400	41.95656500	41.95656500
H	51.00420800	42.12284900	42.12284900
H	52.50797400	41.42616500	41.42616500
C	52.74875000	43.28594700	43.28594700
H	52.36419700	43.73272000	43.73272000
O	54.17180000	43.04892700	43.04892700

C	54.83903400	44.30373200	44.30373200
H	55.02274000	44.72780000	44.72780000
N	56.13948600	44.08387800	44.08387800
C	56.49410400	44.13594800	44.13594800
H	55.76612700	44.36767500	44.36767500
N	57.75996700	43.88878000	43.88878000
C	58.27937300	43.64026400	43.64026400
C	59.58392800	43.32970100	43.32970100
N	60.62129100	43.20870900	43.20870900
H	61.54263000	43.04568800	43.04568800
H	60.49472000	43.41125500	43.41125500
N	59.79809600	43.13823900	43.13823900
C	58.75554600	43.23096100	43.23096100
H	58.97926800	43.04882200	43.04882200
N	57.47324300	43.51066700	43.51066700
C	57.29364700	43.73384800	43.73384800
C	52.69008400	44.32215700	44.32215700
H	52.90214900	43.81860500	43.81860500
O	51.53391900	45.11231500	45.11231500
H	50.75979400	44.57273600	44.57273600
C	53.87714500	45.24683100	45.24683100
H	54.34496900	45.69509100	45.69509100
O	53.45023700	46.25239200	46.25239200
H	52.51760600	46.40827200	46.40827200
C	47.73299500	38.19399500	38.19399500
H	48.72928300	38.54477000	38.54477000
C	46.66471800	39.25639900	39.25639900
H	45.70292800	38.91914400	38.91914400
H	46.89118100	40.17866300	40.17866300
C	46.49622200	39.51953900	39.51953900
H	46.11390200	38.60548600	38.60548600
H	45.72123200	40.28290000	40.28290000
C	47.74869000	39.95529000	39.95529000
H	48.54636900	39.20544900	39.20544900
H	47.47344500	39.99044500	39.99044500
C	48.31979200	41.33648600	41.33648600
H	48.93540300	41.70696100	41.70696100
H	47.52004100	42.06429900	42.06429900
N	49.19822100	41.35760400	41.35760400
H	48.68031600	41.27191800	41.27191800
H	49.95595900	40.63443100	40.63443100
H	49.62047100	42.29244700	42.29244700
C	47.84930000	37.79067800	37.79067800
O	47.91037600	38.63884100	38.63884100
N	47.92926000	36.46929900	36.46929900
H	47.90628700	35.81632000	35.81632000
C	47.96169800	35.94691000	35.94691000
H	47.53882400	36.71803000	36.71803000
H	47.32744700	35.05995700	35.05995700
C	49.31970500	35.57665500	35.57665500
O	49.31010400	34.87280900	34.87280900

N	50.43969500	36.09648400	36.09648400
H	50.35089800	36.81173300	36.81173300
C	51.66000200	36.18100600	36.18100600
H	51.68104900	35.30539000	35.30539000
C	51.67636200	37.49044100	37.49044100
H	50.64354300	37.62239400	37.62239400
C	52.54832900	37.25335800	37.25335800
H	53.59520500	37.07395300	37.07395300
H	52.49001900	38.12927800	38.12927800
H	52.19926300	36.38562000	36.38562000
O	52.09390000	38.67830900	38.67830900
H	53.58038700	39.38597400	39.38597400
O	45.74866000	40.50277700	40.50277700
H	45.36279000	41.19840500	41.19840500
H	45.31814100	40.38763800	40.38763800
O	44.94328600	45.38530500	45.38530500
H	45.71037400	44.88530900	44.88530900
H	44.13759800	44.94430500	44.94430500
O	51.11974700	38.55058300	38.55058300
H	50.43207400	39.09465100	39.09465100
H	51.76939200	38.53480600	38.53480600
O	45.04273600	42.45483800	42.45483800
H	45.02326900	42.38814800	42.38814800
H	45.83160000	43.03262300	43.03262300
O	51.06206100	39.33480500	39.33480500
H	51.20729500	39.03992600	39.03992600
H	51.90203300	39.22292300	39.22292300
O	42.88684900	43.92786600	43.92786600
H	43.56323300	43.35554200	43.35554200
H	42.52446100	43.33783200	43.33783200
O	42.46550300	40.38237600	40.38237600
H	42.30414800	40.79848400	40.79848400
H	43.28063100	39.87282600	39.87282600
O	42.80332600	41.77303100	41.77303100
H	43.68660800	42.12844600	42.12844600
H	42.98070000	40.99743000	40.99743000
H	47.54018400	37.31008500	37.31008500
H	42.10266300	43.55727100	43.55727100
H	52.53409600	36.16311200	36.16311200
H	46.00639000	47.89609000	47.89609000
H	47.20434200	49.37829000	49.37829000
H	48.32323800	38.22933800	38.22933800
H	55.11755600	46.81991700	46.81991700
H	56.06697600	42.17812500	42.17812500
H	44.41758600	45.98215500	45.98215500

Product 1

C	-4.56248300	2.67228500	2.67228500
H	-4.22186300	2.29553900	2.29553900
C	-3.38818100	3.36004100	3.36004100
H	-2.54842300	2.67447300	2.67447300
H	-3.68368000	3.74820400	3.74820400
H	-3.03051400	4.20089800	4.20089800
C	-5.11709300	1.49516300	1.49516300
O	-4.80855100	0.31452200	0.31452200
N	-5.96014000	1.82627900	1.82627900
H	-6.17288000	2.80362700	2.80362700
C	-6.58348400	0.85828900	0.85828900
H	-6.27527700	1.01183500	1.01183500
H	-6.26571400	-0.13274200	-0.13274200
C	6.84651800	-1.16671500	-1.16671500
H	7.81679100	-0.71930300	-0.71930300
H	7.01539200	-1.98836900	-1.98836900
C	6.16449000	-1.67086600	-1.67086600
N	6.15262600	-0.98606600	-0.98606600
H	6.66508200	-0.13149300	-0.13149300
C	5.37300900	-1.62237700	-1.62237700
H	5.12768200	-1.26089200	-1.26089200
N	4.88868800	-2.70751400	-2.70751400
C	5.36446400	-2.76245400	-2.76245400
H	5.08044500	-3.54228800	-3.54228800
C	-1.79348100	-3.57171700	-3.57171700
H	-1.00198200	-3.56038600	-3.56038600
C	-3.13937200	-3.93157300	-3.93157300
H	-3.13418600	-4.99398100	-4.99398100
H	-3.97363100	-3.78179300	-3.78179300
C	-3.34900800	-3.13170600	-3.13170600
O	-2.32492400	-2.97556100	-2.97556100
O	-4.50510300	-2.67209600	-2.67209600
C	-1.90292000	-2.22451700	-2.22451700
O	-2.24166200	-2.15630200	-2.15630200
N	-1.68961300	-1.11562200	-1.11562200
H	-1.45744000	-1.23264000	-1.23264000
C	-1.99456900	0.21634900	0.21634900
H	-2.54586200	0.76047800	0.76047800
H	-2.62236400	0.11486400	0.11486400
C	-0.83488500	1.13248600	1.13248600
O	-1.03476700	2.03510800	2.03510800
N	0.36417300	0.93651900	0.93651900
H	0.39282900	0.42806600	0.42806600
C	1.50896800	1.76324100	1.76324100
H	1.54520000	1.86588500	1.86588500
C	2.81342600	1.09330500	1.09330500
H	2.66692800	0.76229800	0.76229800
H	3.60825900	1.83919500	1.83919500
C	3.25564100	-0.06329700	-0.06329700
O	4.14294700	0.08042900	0.08042900

N	2.61279800	-1.24171500	-1.24171500
H	2.83289600	-1.98071900	-1.98071900
H	1.95267900	-1.43387900	-1.43387900
C	1.46812800	3.22767100	3.22767100
O	2.29184800	4.03387500	4.03387500
N	0.50049500	3.55044300	3.55044300
H	-0.09990000	2.84167600	2.84167600
C	0.41382100	4.88885000	4.88885000
H	0.95219600	5.55761900	5.55761900
H	0.89987600	4.98825000	4.98825000
C	-1.01877300	5.38271700	5.38271700
O	-1.25743600	6.28292100	6.28292100
N	-1.95019300	4.81747000	4.81747000
H	-1.64554800	4.04617100	4.04617100
C	-3.37748100	5.04628800	5.04628800
H	-3.49441900	5.92787800	5.92787800
C	-4.14594000	3.85369700	3.85369700
H	-3.82649600	3.67389300	3.67389300
H	-3.86660800	2.95722400	2.95722400
C	-5.66042200	4.08603000	4.08603000
H	-5.95787600	4.21678200	4.21678200
H	-5.92858500	5.02139900	5.02139900
C	-6.50909700	2.97313700	2.97313700
H	-7.57313500	3.21155500	3.21155500
H	-6.29247700	2.89809300	2.89809300
N	-6.31980900	1.65745800	1.65745800
H	-6.76506400	1.51295200	1.51295200
C	-5.35629200	0.76562300	0.76562300
N	-4.64315400	0.85425700	0.85425700
H	-4.73667200	1.68766900	1.68766900
H	-3.69267100	0.40109400	0.40109400
N	-5.15309400	-0.26602000	-0.26602000
H	-5.98493400	-0.64319300	-0.64319300
H	-4.62972900	-1.04346800	-1.04346800
N	-2.93944900	6.49228200	6.49228200
H	-3.81044000	6.85811000	6.85811000
C	-2.48451700	5.30457200	5.30457200
N	-1.29400300	4.86260900	4.86260900
H	-0.84779000	5.40404800	5.40404800
H	-1.10642000	3.85859200	3.85859200
N	-3.18772500	4.61073000	4.61073000
H	-4.18414300	4.51484600	4.51484600
H	-2.80216500	3.69102000	3.69102000
C	4.86951900	3.46828500	3.46828500
H	4.72311400	3.91438100	3.91438100
H	5.55048100	2.61732800	2.61732800
C	3.55444100	3.04340000	3.04340000
C	3.25960700	3.28653000	3.28653000
H	3.98037500	3.82321100	3.82321100
C	2.05773500	2.87452200	2.87452200
H	1.85749900	3.07892600	3.07892600

C	1.10814800	2.19714500	2.19714500
O	-0.08372000	1.75524900	1.75524900
H	-0.16731300	1.88622400	1.88622400
C	1.37980500	1.95573600	1.95573600
H	0.63254400	1.44608800	1.44608800
C	2.58347700	2.37355900	2.37355900
H	2.77151900	2.17732300	2.17732300
Mg	-1.77275200	-1.80401000	-1.80401000
O	-0.27686100	1.45539800	1.45539800
P	-1.38129400	1.01289700	1.01289700
O	-2.77077000	1.62915200	1.62915200
O	-1.42610700	-0.54510600	-0.54510600
O	-0.90956000	1.66547000	1.66547000
P	-1.01213100	1.02811200	1.02811200
O	-2.09509500	-0.10749000	-0.10749000
O	-1.50639300	2.21649900	2.21649900
O	0.36268400	0.47340800	0.47340800
P	1.47000300	-2.51037400	-2.51037400
O	0.27199700	-2.48941400	-2.48941400
O	1.33621100	-2.38906200	-2.38906200
O	2.61243000	-1.53670800	-1.53670800
C	2.28926300	-0.67460500	-0.67460500
H	1.23003600	-0.42667000	-0.42667000
H	2.53750900	-1.20387100	-1.20387100
C	3.08236300	0.60425600	0.60425600
H	2.83450700	1.09570600	1.09570600
O	4.49716200	0.29993400	0.29993400
C	5.17307200	1.54953400	1.54953400
H	5.31366600	2.01526200	2.01526200
N	6.49599500	1.30947600	1.30947600
C	6.89820000	1.27635000	1.27635000
H	6.19083700	1.41973900	1.41973900
N	8.18033700	1.06652100	1.06652100
C	8.66341300	0.93998300	0.93998300
C	9.96352800	0.72759100	0.72759100
N	11.03537100	0.58249900	0.58249900
H	11.95087800	0.53032200	0.53032200
H	10.93893200	0.73627100	0.73627100
N	10.13826800	0.65323300	0.65323300
C	9.06352000	0.76198000	0.76198000
H	9.25629400	0.67656700	0.67656700
N	7.78088500	0.95580400	0.95580400
C	7.63914600	1.06644000	1.06644000
C	2.93427400	1.61270700	1.61270700
H	2.96499200	1.05880000	1.05880000
O	1.84553100	2.48504900	2.48504900
H	1.02891200	2.07291600	2.07291600
C	4.23366600	2.46320900	2.46320900
H	4.65282100	2.70303900	2.70303900
O	3.99156500	3.64203700	3.64203700
H	3.04069100	3.81921900	3.81921900

C	-2.04048600	-4.37771900	-4.37771900
H	-1.05283400	-4.09206800	-4.09206800
C	-3.08012100	-3.28823800	-3.28823800
H	-4.04082700	-3.58290900	-3.58290900
H	-2.81023400	-2.36337800	-2.36337800
C	-3.27931400	-3.05194400	-3.05194400
H	-3.61459800	-3.98903000	-3.98903000
H	-4.09805700	-2.33505800	-2.33505800
C	-2.06436300	-2.54120200	-2.54120200
H	-1.21765300	-3.23543700	-3.23543700
H	-2.35030700	-2.52744200	-2.52744200
C	-1.58596800	-1.12201000	-1.12201000
H	-1.03967900	-0.70222300	-0.70222300
H	-2.42743300	-0.45601600	-0.45601600
N	-0.63953400	-1.04248200	-1.04248200
H	-1.08460200	-1.08847100	-1.08847100
H	0.10905100	-1.76909400	-1.76909400
H	-0.20404600	-0.10966400	-0.10966400
C	-1.87113000	-4.63820400	-4.63820400
O	-1.66311000	-3.71156800	-3.71156800
N	-1.90829500	-5.92607600	-5.92607600
H	-2.06207200	-6.65202600	-6.65202600
C	-1.85403900	-6.28660200	-6.28660200
H	-2.14040100	-5.39778800	-5.39778800
H	-2.57487400	-7.07976700	-7.07976700
C	-0.50645900	-6.73400600	-6.73400600
O	-0.49460600	-7.32743400	-7.32743400
N	0.62610000	-6.38583600	-6.38583600
H	0.55369500	-5.72273100	-5.72273100
C	1.88651600	-6.39070800	-6.39070800
H	1.91197800	-7.29645200	-7.29645200
C	1.99321600	-5.16103200	-5.16103200
H	1.01649100	-5.00876700	-5.00876700
C	3.02025700	-5.36548700	-5.36548700
H	4.02547900	-5.53488800	-5.53488800
H	3.02168600	-4.49211900	-4.49211900
H	2.75103600	-6.23826200	-6.23826200
O	2.32931300	-3.96357900	-3.96357900
H	4.15172700	-3.28304800	-3.28304800
O	-3.84976900	-2.00541300	-2.00541300
H	-4.27652800	-1.35486000	-1.35486000
H	-4.29197300	-2.11977800	-2.11977800
O	-4.99315800	2.74482600	2.74482600
H	-4.20378500	2.29494400	2.29494400
H	-5.77136600	2.25634400	2.25634400
O	1.25598000	-4.00043800	-4.00043800
H	0.70995000	-3.42736100	-3.42736100
H	2.06599500	-4.09516300	-4.09516300
O	-4.67566100	-0.18044800	-0.18044800
H	-4.69978300	-0.23627800	-0.23627800
H	-3.92397900	0.45378600	0.45378600

O	1.19735800	-3.12969200	-3.12969200
H	1.31586300	-3.45108500	-3.45108500
H	2.07749300	-3.08323100	-3.08323100
O	-6.94263800	1.15047000	1.15047000
H	-6.22059000	0.62035100	0.62035100
H	-7.25482700	0.55792800	0.55792800
O	-7.17276700	-2.21734200	-2.21734200
H	-7.37504800	-1.79428500	-1.79428500
H	-6.33917300	-2.68236900	-2.68236900
O	-6.89066500	-0.88590700	-0.88590700
H	-6.04481700	-0.45575900	-0.45575900
H	-6.62969500	-1.68419000	-1.68419000
H	-2.31024500	-5.30100300	-5.30100300
H	-7.67514700	0.92073600	0.92073600
H	2.72455500	-6.41119700	-6.41119700
H	-3.78704700	5.30706500	5.30706500
H	-2.55986900	6.80951700	6.80951700
H	-1.55335500	-4.30966700	-4.30966700
H	5.38307600	4.20502700	4.20502700
H	6.21402100	-0.42660300	-0.42660300
H	-5.36798500	3.39548500	3.39548500

Table S5. Cartesian coordinates of 5-coordinated.

Reactant 2

C	-4.808807	2.669062	2.669062
H	-4.544389	2.190775	2.190775
C	-3.610439	3.484623	3.484623
H	-2.753125	2.831713	2.831713
H	-3.852570	3.994247	3.994247
H	-3.318005	4.239328	4.239328
C	-5.170450	1.573078	1.573078
O	-4.427062	0.620288	0.620288
N	-6.365782	1.741816	1.741816
H	-6.914593	2.557273	2.557273
C	-6.829815	0.855083	0.855083
H	-6.907879	1.374129	1.374129
H	-6.098261	0.051576	0.051576
C	6.600183	-1.169923	-1.169923
H	7.402762	-0.468112	-0.468112
H	7.060717	-2.051709	-2.051709
C	5.813647	-1.573834	-1.573834
N	5.213963	-0.665775	-0.665775
H	5.208680	0.351326	0.351326
C	4.552025	-1.361947	-1.361947
H	3.994809	-0.868603	-0.868603
N	4.679659	-2.664910	-2.664910
C	5.471220	-2.803246	-2.803246
H	5.763294	-3.773965	-3.773965
C	-2.039815	-3.574919	-3.574919
H	-1.340650	-3.304419	-3.304419
C	-3.240969	-4.360975	-4.360975
H	-2.868520	-5.262485	-5.262485
H	-3.902326	-4.659339	-4.659339
C	-4.033857	-3.509541	-3.509541
O	-3.387066	-3.049908	-3.049908
O	-5.244211	-3.264559	-3.264559
C	-2.522682	-2.332686	-2.332686
O	-3.502354	-2.360492	-2.360492
N	-1.816240	-1.187993	-1.187993
H	-1.118499	-1.204559	-1.204559
C	-2.310575	0.077475	0.077475
H	-3.161146	0.447580	0.447580
H	-2.680025	-0.080916	-0.080916
C	-1.317961	1.237532	1.237532
O	-1.748085	2.383189	2.383189
N	-0.013276	0.934582	0.934582
H	0.240373	-0.045704	-0.045704
C	1.099516	1.883276	1.883276
H	1.348588	2.102275	2.102275
C	2.338965	1.286563	1.286563
H	2.066519	0.998037	0.998037
H	3.101743	2.063986	2.063986

C	2.962896	0.114841	0.114841
O	3.844213	0.292571	0.292571
N	2.436443	-1.110297	-1.110297
H	2.903325	-1.901302	-1.901302
H	1.876878	-1.284291	-1.284291
C	0.833798	3.255348	3.255348
O	1.274489	4.269057	4.269057
N	0.233704	3.241342	3.241342
H	-0.163327	2.389158	2.389158
C	0.084157	4.449135	4.449135
H	0.751452	5.201208	5.201208
H	0.385929	4.276283	4.276283
C	-1.313488	5.055995	5.055995
O	-1.509370	5.953939	5.953939
N	-2.229768	4.619397	4.619397
H	-1.965808	3.873598	3.873598
C	-3.623816	5.043075	5.043075
H	-3.837591	5.400449	5.400449
C	-4.611902	3.942986	3.942986
H	-4.163151	3.343784	3.343784
H	-5.492871	4.438726	4.438726
C	-5.061280	3.048817	3.048817
H	-5.406037	3.687313	3.687313
H	-4.212163	2.479930	2.479930
C	-6.234119	2.133042	2.133042
H	-7.062771	2.724629	2.724629
H	-6.610082	1.630457	1.630457
N	-5.935552	1.080162	1.080162
H	-5.993881	1.320140	1.320140
C	-5.833809	-0.228768	-0.228768
N	-5.607299	-0.604489	-0.604489
H	-4.917201	-0.061077	-0.061077
H	-5.525832	-1.631212	-1.631212
N	-6.017229	-1.161349	-1.161349
H	-6.157926	-0.903682	-0.903682
H	-5.667236	-2.104399	-2.104399
N	-3.185818	6.489084	6.489084
H	-4.165470	6.669400	6.669400
C	-2.901284	5.329475	5.329475
N	-1.655085	4.869010	4.869010
H	-1.073602	5.171528	5.171528
H	-1.414981	4.028879	4.028879
N	-3.814087	4.688565	4.688565
H	-4.755861	5.048791	5.048791
H	-3.689054	3.644607	3.644607
C	4.623182	3.465078	3.465078
H	4.471522	3.830134	3.830134
H	5.311768	2.613120	2.613120
C	3.313008	3.075580	3.075580
C	2.982523	3.470781	3.470781
H	3.671116	4.087751	4.087751

C	1.780803	3.087759	3.087759
H	1.548678	3.414915	3.414915
C	0.880439	2.281145	2.281145
O	-0.297951	1.827785	1.827785
H	-0.498359	2.149002	2.149002
C	1.185721	1.887497	1.887497
H	0.468545	1.284198	1.284198
C	2.383853	2.282684	2.282684
H	2.602072	1.969108	1.969108
M	-2.700828	-1.872810	-1.872810
O	-1.031693	2.340981	2.340981
P	-2.064300	1.394588	1.394588
O	-3.407503	2.037431	2.037431
O	-2.191346	0.001977	0.001977
O	-1.288490	1.068865	1.068865
P	-1.853622	1.086778	1.086778
O	-2.941176	0.012176	0.012176
O	-2.086033	2.450744	2.450744
O	-0.487961	0.505468	0.505468
P	0.166469	-0.994476	-0.994476
O	-0.682078	-1.787614	-1.787614
O	0.386914	-1.533701	-1.533701
O	1.619730	-0.728312	-0.728312
C	1.763592	-0.074071	-0.074071
H	0.834030	-0.133665	-0.133665
H	2.547441	-0.612149	-0.612149
C	2.148001	1.377705	1.377705
H	1.436222	1.855008	1.855008
O	3.479014	1.472451	1.472451
C	3.896143	2.795801	2.795801
H	3.312046	3.521458	3.521458
N	5.300662	2.979315	2.979315
C	6.243664	3.582063	3.582063
H	6.009453	3.822139	3.822139
N	7.376627	3.857366	3.857366
C	7.179932	3.436553	3.436553
C	8.005161	3.492555	3.492555
N	9.270808	3.966393	3.966393
H	9.763007	4.104750	4.104750
H	9.595503	4.418865	4.418865
N	7.522534	3.051522	3.051522
C	6.280412	2.552517	2.552517
H	5.931909	2.194888	2.194888
N	5.408192	2.420369	2.420369
C	5.902728	2.898972	2.898972
C	2.169480	2.205211	2.205211
H	2.101956	1.566854	1.566854
O	1.166975	3.200238	3.200238
H	0.348041	2.823753	2.823753
C	3.548706	2.919734	2.919734
H	4.285351	2.329527	2.329527

O	3.518294	4.221647	4.221647
H	2.579030	4.482126	4.482126
C	-2.286820	-4.380923	-4.380923
H	-1.247447	-4.196428	-4.196428
C	-3.202157	-3.270050	-3.270050
H	-4.206646	-3.402918	-3.402918
H	-2.848889	-2.313228	-2.313228
C	-3.318583	-3.228595	-3.228595
H	-3.668926	-4.208814	-4.208814
H	-4.105795	-2.511332	-2.511332
C	-2.052259	-2.864585	-2.864585
H	-1.189132	-3.457447	-3.457447
H	-2.229344	-3.151973	-3.151973
C	-1.663465	-1.381072	-1.381072
H	-0.983735	-1.218505	-1.218505
H	-2.536230	-0.736347	-0.736347
N	-0.946352	-0.884260	-0.884260
H	-1.576145	-0.629524	-0.629524
H	-0.230357	-1.549702	-1.549702
H	-0.511202	0.032167	0.032167
C	-2.281676	-4.431540	-4.431540
O	-2.369840	-3.389076	-3.389076
N	-2.147620	-5.640168	-5.640168
H	-2.064223	-6.441470	-6.441470
C	-2.130923	-5.868014	-5.868014
H	-2.406896	-4.928236	-4.928236
H	-2.880992	-6.617683	-6.617683
C	-0.802923	-6.367462	-6.367462
O	-0.830045	-7.071243	-7.071243
N	0.326497	-5.987256	-5.987256
H	0.285750	-5.250572	-5.250572
C	1.640183	-6.393919	-6.393919
H	1.475424	-7.255600	-7.255600
C	2.429067	-5.332011	-5.332011
H	1.762631	-4.877090	-4.877090
C	3.615489	-5.989991	-5.989991
H	4.278589	-6.468342	-6.468342
H	4.194290	-5.247339	-5.247339
H	3.270184	-6.748162	-6.748162
O	2.875517	-4.337346	-4.337346
H	3.529426	-3.745725	-3.745725
O	0.904408	-3.435189	-3.435189
H	0.321156	-2.873692	-2.873692
H	1.692192	-3.636676	-3.636676
O	0.793211	-2.831144	-2.831144
H	0.871179	-2.984712	-2.984712
H	1.623023	-3.150888	-3.150888
H	-2.574209	-5.356848	-5.356848
H	-7.806709	0.423486	0.423486
H	2.248410	-6.711414	-6.711414
H	-3.748798	5.905261	5.905261

H	-2.588736	6.662581	6.662581
H	-1.499813	-4.208568	-4.208568
H	5.128502	4.253736	4.253736
H	5.956598	-0.693327	-0.693327
H	-5.669161	3.326257	3.326257
O	-4.023405	-1.105341	-1.105341
H	-3.432562	-0.804447	-0.804447
H	-4.334846	-0.251436	-0.251436

Transition State 2

C	45.211006	45.243984	50.148003
H	45.454327	44.701313	51.069077
C	46.423267	46.090038	49.727459
H	47.292782	45.450916	49.559921
H	46.219162	46.645473	48.805580
H	46.676483	46.806103	50.513195
C	44.877655	44.199768	49.094513
O	45.674244	43.324242	48.757271
N	43.640636	44.309616	48.530392
H	43.040943	45.063396	48.826782
C	43.190002	43.430004	47.472004
H	43.031902	43.976261	46.534779
H	43.968956	42.683090	47.318935
C	56.619999	41.404999	59.732998
H	57.673805	41.343910	59.440441
H	56.471481	40.768082	60.606770
C	55.715279	40.961121	58.618080
N	55.658642	41.606640	57.387543
H	56.155720	42.453541	57.059471
C	54.774193	40.991978	56.596745
H	54.509056	41.319042	55.605698
N	54.251225	39.967316	57.260269
C	54.816570	39.928394	58.523228
H	54.530209	39.188198	59.251156
C	47.980000	39.000004	59.145996
H	48.626564	39.306858	58.319321
C	46.778275	38.171329	58.638157
H	47.160053	37.326328	58.054504
H	46.211044	37.789471	59.488731
C	45.844315	39.016613	57.780239
O	46.285206	39.427189	56.639114
O	44.722015	39.317249	58.232265
C	47.537777	40.209480	59.970455
O	47.116653	40.098518	61.115894
N	47.645496	41.422947	59.327812
H	47.847752	41.446629	58.327240
C	47.131981	42.638111	59.923519
H	46.301704	43.054077	59.342590
H	46.748405	42.373497	60.914604
C	48.140602	43.778770	60.107510
O	47.733234	44.926838	60.324051

N	49.440022	43.422451	60.049511
H	49.619434	42.439053	59.877666
C	50.576073	44.260056	60.433910
H	50.559486	44.447041	61.512596
C	51.879032	43.503548	60.089268
H	51.886494	43.261063	59.021114
H	52.715252	44.168407	60.316952
C	52.031155	42.256023	60.963531
O	52.346016	42.343849	62.145920
N	51.738331	41.075562	60.354984
H	51.761868	40.254868	60.943329
H	51.532448	40.950424	59.358654
C	50.624859	45.659725	59.788708
O	51.138874	46.588356	60.406513
N	50.155857	45.750671	58.520397
H	49.750164	44.946484	58.021805
C	50.155228	46.994419	57.794537
H	50.750599	47.722057	58.355873
H	50.611652	46.863022	56.807667
C	48.770596	47.604050	57.556019
O	48.636295	48.475601	56.669022
N	47.779160	47.173683	58.349438
H	47.987732	46.444439	59.035576
C	46.396000	47.617996	58.243999
H	46.343605	48.297482	57.389591
C	45.409863	46.456581	58.077606
H	45.459572	45.821426	58.970257
H	44.399212	46.893253	58.047401
C	45.679276	45.629009	56.815819
H	45.937908	46.293320	55.984081
H	46.560272	44.992985	56.947819
C	44.484528	44.808861	56.327549
H	43.653831	45.475121	56.060295
H	44.772968	44.259533	55.428978
N	43.937641	43.845757	57.296745
H	43.339485	44.201202	58.027458
C	44.155483	42.519619	57.275509
N	45.108738	42.008511	56.478821
H	46.023979	42.472813	56.426693
H	45.249550	40.990803	56.549931
N	43.410393	41.682606	57.997559
H	42.600105	42.007076	58.500099
H	43.770966	40.711323	58.121807
N	46.834000	49.064007	54.445999
H	45.849873	49.189827	54.635735
C	47.188919	47.845470	53.942276
N	48.446064	47.437191	54.059349
H	48.973850	47.803318	54.851681
H	48.713215	46.543179	53.630386
N	46.316414	47.082909	53.270351
H	45.368851	47.414734	53.175976

H	46.488224	46.039837	53.361240
C	54.642998	46.040001	47.281002
H	54.413742	46.434410	46.285286
H	55.274380	45.154102	47.140141
C	53.377342	45.704536	48.048389
C	53.174446	46.108849	49.373978
H	53.930264	46.703438	49.881535
C	52.014725	45.774269	50.084644
H	51.900154	46.105888	51.111217
C	51.022408	45.006294	49.469948
O	49.873810	44.590134	50.090103
H	49.736919	44.860172	51.042004
C	51.198215	44.608074	48.136986
H	50.411743	44.037254	47.651749
C	52.355694	44.953129	47.446659
H	52.466976	44.631916	46.413357
M	47.689465	40.109257	55.396263
O	49.151413	44.856281	52.679935
P	48.112629	43.867912	53.305492
O	46.922169	44.578617	53.946774
O	47.671417	42.726288	52.349583
O	49.003231	43.040783	54.436161
P	49.001820	42.855835	56.075096
O	47.839256	41.865833	56.389786
O	48.864365	44.190449	56.750755
O	50.378662	42.140369	56.321125
P	50.952625	40.338017	56.240734
O	49.703148	39.814899	55.470665
O	51.160519	40.240845	57.729137
O	52.176098	40.859741	55.252956
C	51.981770	41.776165	54.171516
H	50.923523	41.973427	54.004982
H	52.399925	41.307793	53.272186
C	52.670021	43.100086	54.430290
H	52.398560	43.460659	55.428329
O	54.115685	42.941357	54.338707
C	54.618732	44.259930	54.164700
H	54.509792	44.840878	55.091412
N	56.034889	44.229866	53.831261
C	56.615685	44.570744	52.614735
H	56.013252	44.804714	51.751301
N	57.922832	44.608185	52.634663
C	58.242645	44.284668	53.937431
C	59.486443	44.202950	54.596157
N	60.654491	44.401264	53.951118
H	61.505905	44.432224	54.488506
H	60.646797	44.718548	52.995213
N	59.507900	43.911041	55.911564
C	58.341644	43.689896	56.521023
H	58.411156	43.447857	57.580269
N	57.099323	43.719013	56.012032

C	57.097637	44.044228	54.702759
C	52.322502	44.200272	53.401867
H	51.888474	43.764046	52.494572
O	51.468754	45.192287	53.920498
H	50.560371	45.054638	53.530098
C	53.703945	44.850430	53.070164
H	54.031059	44.480465	52.093475
O	53.654278	46.249287	53.048859
H	52.791939	46.456844	53.464375
C	47.732998	38.194000	51.739002
H	48.779465	38.361332	51.446358
C	46.906406	39.454411	51.470505
H	45.942520	39.375919	51.980804
H	47.403915	40.311131	51.920341
C	46.654667	39.707588	49.973907
H	46.149483	38.825726	49.556335
H	45.946568	40.538979	49.874241
C	47.884010	40.013054	49.092190
H	48.720596	39.344166	49.330250
H	47.612434	39.794315	48.052322
C	48.379829	41.462944	49.082172
H	49.079266	41.592327	48.249653
H	47.552753	42.164520	48.947273
N	49.109207	41.880527	50.320717
H	48.465782	42.117256	51.147575
H	49.818977	41.181454	50.611572
H	49.565586	42.794010	50.149109
C	47.773079	37.715534	53.175980
O	47.641994	38.471851	54.147278
N	47.995552	36.393063	53.354866
H	48.103859	35.819370	52.531708
C	47.998234	35.740517	54.657269
H	47.387535	36.345364	55.331772
H	47.517982	34.763134	54.567104
C	49.340553	35.515385	55.377796
O	49.315250	34.850395	56.409592
N	50.457077	36.070621	54.844437
H	50.366955	36.768444	54.109463
C	51.660000	36.181004	55.671997
H	51.723385	35.269337	56.268127
C	51.609295	37.427589	56.618492
H	50.568539	37.516285	56.958687
C	52.479881	37.182068	57.856552
H	53.531609	37.033531	57.577503
H	52.398014	38.043949	58.523838
H	52.147961	36.292542	58.402630
O	51.999580	38.625340	55.941860
H	53.468441	39.390163	56.867905
O	50.974010	38.576424	53.441822
H	50.287949	39.027050	53.991051
H	51.676514	38.553471	54.144653

O	50.945801	39.840801	51.092537
H	51.034546	39.401985	51.981911
H	51.689579	39.522980	50.568462
H	47.372025	37.367985	51.110565
H	42.256664	42.925205	47.744659
H	52.538643	36.245525	55.022198
H	46.133690	48.197193	59.139019
H	47.434971	49.368908	55.211742
H	48.578388	38.369522	59.809860
H	55.240582	46.789364	47.807819
H	56.404797	42.436291	60.030678
H	44.343121	45.883121	50.354534
O	46.418415	41.080765	54.003796
H	46.846741	41.693737	53.330158
H	45.746101	41.621208	54.443367

Product 2

C	-4.80880600	2.66906300	-4.91572400
H	-4.50722300	2.14117500	-4.00362500
C	-3.65157100	3.56610500	-5.38534800
H	-2.75855700	2.96796500	-5.57856700
H	-3.91368000	4.10549300	-6.30238100
H	-3.40603900	4.29749200	-4.61154000
C	-5.14508100	1.61721900	-5.95949700
O	-4.35133800	0.73763500	-6.29431600
N	-6.38082900	1.72949100	-6.52825600
H	-6.97887500	2.48488700	-6.23261600
C	-6.82981500	0.85508300	-7.59172500
H	-6.98717300	1.40595200	-8.52650100
H	-6.04971700	0.10968600	-7.74707900
C	6.60018200	-1.16992400	4.66927100
H	7.54010900	-0.67207100	4.41182900
H	6.83317200	-2.01851600	5.31600300
C	5.87735300	-1.63654500	3.45449200
N	5.72044800	-0.85830600	2.31513900
H	6.09968100	0.08216700	2.09682200
C	4.96543900	-1.50139700	1.42588500
H	4.65647400	-1.09058200	0.47786200
N	4.63090600	-2.68003100	1.94509900
C	5.18461500	-2.78961500	3.20948500
H	5.03458500	-3.65647200	3.82989200
C	-2.03981600	-3.57492000	4.08227100
H	-1.37034500	-3.36852700	3.24520600
C	-3.28772600	-4.34394000	3.61194200
H	-2.97775000	-5.33352600	3.25549400
H	-3.98930400	-4.47052700	4.43873900
C	-3.95528400	-3.59742400	2.46609400
O	-3.21553300	-3.36479200	1.45094500
O	-5.14813200	-3.22078600	2.55382000
C	-2.41743500	-2.29498800	4.83518400
O	-2.95059700	-2.34763700	5.94087600

N	-2.12953800	-1.11123600	4.20037300
H	-1.91945000	-1.11161700	3.19841500
C	-2.58360500	0.15207000	4.74928000
H	-3.36415000	0.59645900	4.12262500
H	-3.01583600	-0.05368700	5.73260600
C	-1.53075000	1.25299000	4.92554500
O	-1.90122900	2.39175700	5.23656900
N	-0.24589400	0.89015500	4.73718300
H	-0.08744400	-0.06659400	4.44710200
C	0.92218900	1.74414400	4.94709300
H	1.10615500	1.88086800	6.01802300
C	2.16913500	1.10125800	4.27991300
H	1.88295200	0.78581200	3.26955000
H	2.94148900	1.87080600	4.22449100
C	2.77242800	-0.04523700	5.08656500
O	3.68230200	0.13844600	5.89722000
N	2.20856300	-1.26687600	4.88710700
H	2.61020800	-2.02152100	5.42545000
H	1.71148900	-1.52447400	4.03103500
C	0.80942800	3.17673900	4.38735500
O	1.36097100	4.09843000	4.98376900
N	0.20409400	3.30081500	3.18268100
H	-0.29768500	2.52969200	2.72144500
C	0.12046300	4.56959000	2.51312500
H	0.72916100	5.29091200	3.06778500
H	0.52258800	4.49384900	1.49636400
C	-1.28874900	5.14274100	2.38293900
O	-1.47891900	6.06604400	1.56084700
N	-2.22872900	4.63246700	3.18874600
H	-1.96203800	3.87256500	3.81823600
C	-3.62381600	5.04307500	3.18027200
H	-3.73808000	5.76835800	2.37019800
C	-4.60678500	3.88328800	3.01402700
H	-4.43749700	3.16928700	3.83020100
H	-5.61902500	4.29619900	3.15375200
C	-4.48184800	3.18019600	1.66115800
H	-4.49348800	3.92540300	0.85514000
H	-3.51622000	2.66718600	1.58966200
C	-5.62451700	2.21140100	1.35807400
H	-6.55763700	2.75951900	1.16735800
H	-5.38543300	1.63210200	0.46446600
N	-5.92573600	1.26349300	2.44264900
H	-6.39994400	1.62868100	3.25553200
C	-5.70739400	-0.05977000	2.41576900
N	-4.87296100	-0.60528700	1.51713600
H	-3.90758400	-0.22697400	1.42837700
H	-4.88685600	-1.63323000	1.56235800
N	-6.35160900	-0.85980400	3.27622500
H	-7.15562700	-0.53884300	3.79024000
H	-6.05401500	-1.84104500	3.32513900
N	-3.18581800	6.48908400	-0.61772700

H	-4.17522700	6.60989800	-0.45403000
C	-2.82857500	5.29955800	-1.17931200
N	-1.56283800	4.90562900	-1.09506100
H	-1.01372600	5.29461200	-0.33403200
H	-1.28469300	4.02201700	-1.54012500
N	-3.69752100	4.55469800	-1.87549300
H	-4.65688400	4.86658100	-1.89510000
H	-3.51836100	3.50179600	-1.81686400
C	4.62318000	3.46507800	-7.78272500
H	4.42982300	3.89362400	-8.77193300
H	5.21287300	2.55252800	-7.93610200
C	3.33081700	3.17632000	-7.04185400
C	3.14436600	3.52449800	-5.69794700
H	3.93198800	4.04291600	-5.15624700
C	1.95996900	3.23113100	-5.01176600
H	1.85787500	3.51747700	-3.97094200
C	0.92303700	2.56413600	-5.66954700
O	-0.25335200	2.19844100	-5.07485200
H	-0.35938900	2.40118100	-4.09667600
C	1.08296500	2.22459600	-7.02117100
H	0.26379400	1.73400900	-7.53924200
C	2.26674400	2.52700900	-7.68702800
H	2.36504400	2.25361200	-8.73544300
M	-2.09643500	-2.23090500	0.27822600
O	-0.85346600	2.36231500	-2.47220000
P	-1.87539100	1.35371700	-1.83382200
O	-3.15897900	2.03206000	-1.35408000
O	-2.18522300	0.13311700	-2.75519000
O	-1.03700000	0.67029500	-0.60796100
P	-1.07281500	0.62025600	1.08197200
O	-2.11454000	-0.55547700	1.34008200
O	-1.59663900	1.93949000	1.61459400
O	0.33222900	0.20987400	1.47005400
P	1.21538500	-2.59446800	1.10995800
O	-0.05697500	-2.67752400	0.25597200
O	1.13799100	-2.48259000	2.59583800
O	2.30584800	-1.65758000	0.35066300
C	1.96206500	-0.75848000	-0.73197200
H	0.88674900	-0.59638000	-0.75977900
H	2.30034500	-1.21759000	-1.66949600
C	2.61907500	0.58613800	-0.52770400
H	2.31932000	0.97884900	0.44880300
O	4.07281000	0.45449600	-0.60447000
C	4.54375100	1.77620100	-0.83393300
H	4.38596600	2.39960000	0.05708400
N	5.97021100	1.77929900	-1.12026600
C	6.57934500	2.09749900	-2.32897900
H	5.99891900	2.25022400	-3.22521200
N	7.87986900	2.21988600	-2.25985500
C	8.16357000	1.98627500	-0.92929400
C	9.37779300	2.02770800	-0.21414800

N	10.56036700	2.26660200	-0.81953200
H	11.37430200	2.41205700	-0.24372000
H	10.57074700	2.55307600	-1.78538700
N	9.35705500	1.81346900	1.11631200
C	8.18194800	1.54487900	1.68882900
H	8.21660100	1.37181500	2.76306000
N	6.96605800	1.45498800	1.12489800
C	7.00404300	1.71069400	-0.19895600
C	2.26286600	1.62984200	-1.61793500
H	1.83318500	1.14387000	-2.50184100
O	1.41230400	2.65106000	-1.16182500
H	0.51480000	2.52067800	-1.58667000
C	3.64399400	2.27217700	-1.98120500
H	3.99316800	1.83952700	-2.92456200
O	3.57689700	3.66486500	-2.09639600
H	2.71012500	3.88240000	-1.69143400
C	-2.28681900	-4.38092500	-3.32472500
H	-1.25701000	-4.33825900	-3.70718300
C	-3.01635500	-3.06850300	-3.63722400
H	-3.97507600	-3.05828200	-3.11142300
H	-2.44704000	-2.23959200	-3.21905700
C	-3.26688200	-2.84608200	-5.13822600
H	-3.76554200	-3.73704600	-5.54396800
H	-3.98084500	-2.02195600	-5.25319300
C	-2.03273400	-2.53856000	-6.01462500
H	-1.16539500	-3.13756600	-5.70741100
H	-2.25991600	-2.84761600	-7.04202100
C	-1.61978200	-1.06467600	-6.11268400
H	-0.93471600	-0.94240600	-6.95854500
H	-2.48716900	-0.42111600	-6.27726900
N	-0.90765000	-0.54177800	-4.90827300
H	-1.52666500	-0.34777500	-4.04085500
H	-0.12822000	-1.16268200	-4.62935500
H	-0.53939200	0.40653900	-5.10502800
C	-2.16856600	-4.69868000	-1.86200900
O	-2.04436800	-3.80519200	-1.02574900
N	-2.14053200	-6.00316100	-1.49613200
H	-2.24470000	-6.70312700	-2.21536400
C	-2.11724300	-6.42504400	-0.10182700
H	-2.55740900	-5.62047000	0.49627900
H	-2.74182600	-7.31298600	0.01910400
C	-0.76048800	-6.74554500	0.54009500
O	-0.75327900	-7.29835600	1.63599500
N	0.37264900	-6.37177200	-0.11828000
H	0.30241800	-5.74195300	-0.91163000
C	1.64018300	-6.39391500	0.60827000
H	1.67444100	-7.32484500	1.17816200
C	1.77649800	-5.20946400	1.57537600
H	0.81740300	-5.06353700	2.07808200
C	2.84294400	-5.44568300	2.63719300
H	3.83103700	-5.60649700	2.18754800

H	2.87475900	-4.58691200	3.31139800
H	2.59497900	-6.33157200	3.22948600
O	2.09690500	-4.01594200	0.79791100
H	3.93668600	-3.29560100	1.51567500
O	0.92634800	-3.98515000	-1.91745600
H	0.38265800	-3.48292200	-1.25620600
H	1.76126200	-4.07599300	-1.42996400
O	1.14321700	-2.38370100	-4.12740900
H	1.07482500	-2.96050100	-3.33082300
H	1.80983500	-2.78806700	-4.69360900
H	-2.76969100	-5.21836700	-3.84729300
H	-7.76307200	0.34759100	-7.32330200
H	2.46671500	-6.39148400	-0.10941000
H	-3.84543200	5.57120400	4.11771100
H	-2.60731300	6.73776600	0.18988600
H	-1.49329100	-4.19277000	4.80212300
H	5.24925600	4.17098100	-7.22940000
H	5.96703300	-0.47733300	5.23702400
H	-5.69238600	3.27378400	-4.67434900
O	-3.49223400	-1.39276100	-1.07708400
H	-3.05360800	-0.83222600	-1.79388800
H	-4.10182800	-0.78382000	-0.63683600

Table S6. Cartesian coordinates of 4-coordinated AMPylation.

Reactant 3

C	-4.27155300	3.40515700	-7.15548600
C	-3.66204200	2.05002100	-6.78252500
O	-2.49285700	1.75991700	-7.04512100
C	-3.40384100	4.62654200	-6.77949500
C	-2.13631200	4.73650100	-7.64863700
C	-3.10903900	4.64101500	-5.27357200
C	-1.34304700	6.03142800	-7.44514500
H	-4.43027900	3.38963800	-8.24106500
H	-4.03132200	5.50220600	-7.00763000
H	-2.43426200	4.66368500	-8.70288800
H	-1.49860200	3.86653400	-7.45422300
H	-2.63258700	5.57729300	-4.97011700
H	-4.03298100	4.53604000	-4.69337600
H	-2.44026500	3.81899700	-4.99557600
H	-0.51072000	6.08838200	-8.15308800
H	-1.97200100	6.91580700	-7.60387700
H	-0.91746300	6.09998500	-6.43890600
N	-4.48852100	1.16898900	-6.16118200
C	-4.09054600	-0.19887900	-5.82846000
H	-5.46532200	1.41278800	-6.07166000
H	-3.02844300	-0.16951400	-5.57290400
C	7.36844700	-2.38286000	4.39952400
C	7.75213700	-3.20345400	3.15111700
C	6.81504400	-3.05773600	2.00225700
N	6.52642400	-1.86190100	1.39019600
C	6.07031300	-3.99560000	1.27323600
C	5.66032800	-2.09549000	0.35954600
N	5.37427000	-3.39240000	0.27431000
H	7.29526600	-1.31295100	4.17678000
H	7.81254700	-4.26598700	3.40369300
H	8.75966000	-2.90882400	2.82305700
H	6.90072900	-0.95886700	1.64588800
H	6.03285400	-5.06295900	1.43985100
H	5.27525200	-1.30717300	-0.28102100
C	-0.74455100	-5.13786100	2.96152400
C	-1.28046200	-3.91773400	3.72127100
O	-2.19502800	-3.99313300	4.53579200
C	-1.89255400	-5.91131900	2.27757100
C	-2.58547000	-5.06328800	1.20382600
O	-1.89144100	-4.83078800	0.14075100
O	-3.72991600	-4.63314500	1.41658100
H	0.01821200	-4.84807800	2.23533900
H	-2.63001500	-6.20802700	3.02432500
H	-1.48035800	-6.80383100	1.79587400
N	-0.67185400	-2.73944900	3.39424000
C	-1.13475100	-1.46173400	3.89086100
C	-0.03882300	-0.43252400	4.17041400
O	-0.31147600	0.60414700	4.77056100

H	0.01684200	-2.77279400	2.64232500
H	-1.67799500	-1.63306400	4.82330400
H	-1.82223100	-0.97929100	3.18546700
N	1.21468800	-0.70063700	3.70428600
C	2.35515500	0.13107800	4.01825000
C	2.37391500	1.53391100	3.33932000
O	3.09143200	2.40532300	3.80830900
C	3.65227500	-0.59405200	3.62579100
C	3.82329500	-1.91051400	4.39072600
O	4.16110000	-1.91628800	5.56765000
N	3.61567000	-3.04874500	3.66861900
H	1.35773600	-1.47487500	3.05509300
H	2.35577900	0.34893600	5.08980200
H	4.48506800	0.05898600	3.89531400
H	3.67502200	-0.76771000	2.54353100
H	3.59239300	-3.90537200	4.20661500
H	3.08838000	-3.03206400	2.79537500
N	1.61698300	1.68313500	2.22140400
C	1.61031800	2.92144400	1.46204200
C	0.24065800	3.59343700	1.35087500
O	0.04565000	4.41872500	0.44999400
H	1.10817300	0.88210300	1.86143500
H	1.94968200	2.76003700	0.43401100
H	2.31096000	3.60792000	1.94457800
N	-0.69727300	3.24992400	2.24734900
C	-2.10955000	3.57113800	2.03952400
C	-2.96250200	2.81972400	3.06999000
C	-4.44155400	2.67227300	2.65125700
C	-5.06059800	1.34250700	3.09941600
N	-4.44069000	0.23560900	2.36516000
C	-4.84921500	-1.03637100	2.39654900
N	-5.82133800	-1.41574100	3.25901900
N	-4.34520100	-1.91929100	1.54179800
H	-0.47502500	2.51254500	2.90526000
H	-2.38548000	3.26136600	1.02604100
H	-2.89428400	3.30623700	4.04920500
H	-2.52351600	1.82593400	3.19265400
H	-4.53633100	2.73254400	1.56010000
H	-5.04751100	3.48602400	3.06264100
H	-6.13964700	1.34150700	2.90532500
H	-4.91456800	1.21643100	4.18289200
H	-3.58697800	0.42341100	1.82004100
H	-6.07862800	-2.39072200	3.29261100
H	-5.97361700	-0.88258200	4.10032800
H	-4.38379700	-2.93197300	1.68200100
H	-3.66328200	-1.62567400	0.84012700
C	-0.53955100	7.87014000	0.95252600
C	0.14311400	7.49009400	-0.36402000
C	-0.82180600	7.29446200	-1.54313300
N	-1.86790100	6.27828900	-1.35324800
C	-1.83917400	5.01631800	-1.81862100

N	-0.69369400	4.48815400	-2.26883600
N	-2.97636400	4.31299400	-1.87892400
H	-1.14936000	8.77577500	0.84910700
H	-1.18082800	7.05763500	1.30953000
H	0.72986600	6.58043600	-0.21367100
H	0.84027000	8.28255100	-0.66395300
H	-0.27146500	7.01858300	-2.44648800
H	-1.34259800	8.23551600	-1.75526800
H	-2.67142100	6.53685400	-0.80050600
H	-0.73599800	3.56189000	-2.71143500
H	0.11591700	4.63412400	-1.67381800
H	-2.96698700	3.28884600	-2.03396000
H	-3.86315100	4.79193700	-1.86919800
Mg	-1.49076100	-3.30940800	-0.96194900
P	-1.61521900	0.83833500	-2.62301000
O	-0.60888500	1.79972500	-3.26579700
O	-2.89614800	1.53348400	-2.16414300
O	-1.84361500	-0.45891900	-3.40305800
P	-1.29817300	-0.35673400	0.10834600
O	-2.13765100	-1.59274600	-0.25525400
O	-1.89211500	0.54554100	1.14712800
O	-0.78091100	0.37861000	-1.21285200
P	1.14912200	-2.13503900	0.29509900
O	0.43206900	-2.97750400	-0.74546100
O	1.55475300	-2.74288100	1.62038700
O	0.15597400	-0.86512200	0.70763600
O	2.43387900	-1.42120000	-0.31539300
C	2.36271900	-0.79733000	-1.62816600
C	3.15331400	0.48417200	-1.55495700
O	4.55482200	0.18703200	-1.49297300
C	2.94102500	1.41783500	-2.76694300
O	1.94558900	2.38987000	-2.54271300
C	4.31930600	2.09506100	-2.88129200
O	4.40598100	3.15011900	-1.94013900
C	5.25944900	0.97413800	-2.46447500
H	1.32459000	-0.57784100	-1.88642200
H	2.79073300	-1.50378800	-2.34294000
H	2.86475300	1.03014800	-0.64882700
H	2.73601700	0.83391600	-3.67588500
H	1.06406800	2.06425300	-2.83829200
H	4.52680800	2.44793500	-3.90182600
H	3.55661900	3.61589300	-1.99895800
H	6.17349600	1.36944300	-2.01182000
O	-1.99964900	-2.95870200	-2.86283000
H	-1.96523000	-1.95935100	-3.07670100
H	-1.73635000	-3.43944100	-3.65526300
C	-4.91617800	-0.72691600	-4.65967500
H	-5.98801200	-0.73435600	-4.89247300
H	-4.62119800	-1.75506800	-4.43308900
H	-4.74554100	-0.12143500	-3.76613400
H	-0.27914600	-5.79296500	3.70668400

H	8.14119200	-2.50038100	5.16395600
H	6.41756300	-2.70642300	4.82438300
H	5.53005300	0.34883200	-3.32695900
H	-5.25892600	3.49641700	-6.68684800
H	-4.20423300	-0.84427500	-6.71085900
H	-2.25583700	4.65515100	2.11102800
H	0.20356600	8.06050300	1.73166500
C	2.18065400	-5.09022700	-3.27978600
H	1.78972800	-6.09181100	-3.48558700
C	3.27939000	-5.14248100	-2.22566900
H	2.85942300	-5.55911700	-1.29336800
C	4.46571800	-6.02212800	-2.65260100
H	4.90158000	-5.63739600	-3.57957700
H	5.24502100	-6.02665200	-1.88398600
H	4.14244100	-7.05501600	-2.81717600
O	3.71012200	-3.80860000	-1.98971100
H	4.28500400	-3.79185000	-1.19111500
H	2.57034800	-4.67163800	-4.21417500
H	1.36472900	-4.45379000	-2.92686600

Transition State 3

C	-4.27155200	3.40514900	-7.15548100
C	-3.64705600	2.05682000	-6.77048500
O	-2.46805100	1.78436700	-7.00404000
C	-3.35192200	4.63305500	-6.98237200
C	-2.22042600	4.67109000	-8.02995700
C	-2.84175100	4.74692300	-5.53829100
C	-1.42739400	5.98190100	-8.05130000
H	-4.57334800	3.32194500	-8.20798500
H	-3.99830800	5.50313600	-7.17658100
H	-2.66473800	4.51143500	-9.02152200
H	-1.54982800	3.82318200	-7.85687600
H	-2.30537100	5.68700300	-5.38074500
H	-3.67271100	4.71863600	-4.82391900
H	-2.16176200	3.92449600	-5.29384900
H	-0.69821000	5.97851300	-8.86731500
H	-2.08520000	6.84667800	-8.20080100
H	-0.87207500	6.14265400	-7.12164100
N	-4.48511100	1.16834300	-6.17424300
C	-4.09054600	-0.19887100	-5.82846500
H	-5.46034800	1.41565600	-6.08493100
H	-3.01015100	-0.17466000	-5.67037700
C	7.36844700	-2.38286000	4.39952400
C	7.88617400	-3.16259000	3.17469900
C	6.97270700	-3.07421200	1.99369800
N	6.84655100	-1.92756100	1.21306100
C	6.05885300	-3.94988500	1.47898900
C	5.90044800	-2.09360000	0.27541200
N	5.41990600	-3.32414600	0.42528700
H	7.23315300	-1.32025500	4.17264000
H	8.00437500	-4.22039900	3.42764900

H	8.88287400	-2.80397200	2.88869900
H	7.38604500	-1.08051300	1.32948900
H	5.80636900	-4.94999200	1.78922300
H	5.56671700	-1.35747000	-0.45627000
C	-0.74455100	-5.13786100	2.96152400
C	-1.21297100	-4.00325500	3.85953600
O	-2.00086200	-4.16786600	4.78256600
C	-1.93409600	-5.82863200	2.25939000
C	-2.51873400	-4.93667500	1.15900300
O	-1.72689500	-4.69929400	0.16860800
O	-3.66627000	-4.48237400	1.28603000
H	-0.00467300	-4.79786900	2.23495500
H	-2.70764200	-6.07491400	2.98865100
H	-1.57981200	-6.75159100	1.78824600
N	-0.70265100	-2.76000900	3.55113400
C	-1.11576800	-1.58073900	4.27801500
C	0.10748500	-0.81149600	4.80208700
O	0.16801900	-0.37080800	5.93851000
H	-0.14371000	-2.66794500	2.70376500
H	-1.72079400	-1.89867300	5.12596600
H	-1.68644500	-0.89712100	3.63435200
N	1.10954200	-0.68053700	3.87539600
C	2.32321300	0.03305500	4.13670100
C	2.30315000	1.50398000	3.57060900
O	2.89697900	2.37851500	4.18100500
C	3.52036700	-0.72471000	3.52516900
C	3.74449800	-2.02571800	4.30547800
O	4.29104200	-2.00141300	5.40512500
N	3.31131300	-3.16109600	3.70981300
H	0.85509000	-0.83880000	2.87085700
H	2.44775000	0.14412600	5.21547400
H	4.41235800	-0.10421400	3.64412100
H	3.34543200	-0.91938800	2.46180000
H	3.33418200	-3.99201000	4.28523700
H	2.73463800	-3.17719300	2.86145900
N	1.64170800	1.67667600	2.39866000
C	1.65556600	2.93914700	1.68706000
C	0.30258300	3.67240000	1.62618600
O	0.24420900	4.72959300	0.98919900
H	1.26261600	0.85470500	1.90294800
H	1.95245100	2.77684700	0.64586300
H	2.37037300	3.61235700	2.16399000
N	-0.74251800	3.12120600	2.27254200
C	-2.10955000	3.57113800	2.03952400
C	-3.06549900	3.01864900	3.11115100
C	-4.51276200	2.83855300	2.58655600
C	-5.16307400	1.51249200	3.00315300
N	-4.46340400	0.38489600	2.38632500
C	-4.95351100	-0.84990800	2.26411500
N	-6.14627800	-1.17072700	2.82758900
N	-4.30399300	-1.75490800	1.54059700

H	-0.68477500	2.12789000	2.49196300
H	-2.42233400	3.21308400	1.04772700
H	-3.05457200	3.66225200	3.99707500
H	-2.67734600	2.04991500	3.43536700
H	-4.52299400	2.87889300	1.49137600
H	-5.15677700	3.65106700	2.93882700
H	-6.21041400	1.50040700	2.67911400
H	-5.15246800	1.42129000	4.10037600
H	-3.47976200	0.51168900	2.07550500
H	-6.48014600	-2.11808700	2.73967800
H	-6.47609600	-0.65041800	3.62440200
H	-4.46576700	-2.75733800	1.61320800
H	-3.42850100	-1.51392500	1.05947600
C	-0.53955100	7.87014000	0.95252600
C	0.25081100	7.69429700	-0.34298000
C	-0.63410500	7.65102900	-1.59848600
N	-1.77643300	6.73070500	-1.51873700
C	-1.73253400	5.40887300	-1.76970800
N	-0.56203400	4.80113800	-1.98268900
N	-2.87004600	4.71290300	-1.87166500
H	-1.16561900	8.77075200	0.92601900
H	-1.17490800	7.00254900	1.14338400
H	0.85342700	6.78455600	-0.26126200
H	0.95577100	8.52353400	-0.47884800
H	-0.05015500	7.37763900	-2.48267300
H	-1.06013600	8.64129300	-1.78690200
H	-2.63215300	7.08718600	-1.12109400
H	-0.57149100	3.80219500	-2.25667600
H	0.22586600	5.09662800	-1.42224100
H	-2.86604000	3.68511700	-1.71402000
H	-3.75058500	5.19880300	-1.93470000
Mg	-1.22338500	-3.05944400	-0.73403600
P	-1.55114200	1.24116700	-1.72318400
O	-0.50826600	2.09779300	-2.47033300
O	-2.67953300	2.08818400	-1.10666900
O	-2.10562900	0.09095700	-2.57417000
P	-0.96768200	-0.30044600	0.82344700
O	-1.78005300	-1.52694800	0.32215400
O	-1.76348500	0.50059200	1.85660100
O	-0.64272000	0.62134900	-0.49594600
P	1.91168700	-3.23236300	-0.06458800
O	0.71397000	-3.18677400	-1.00670400
O	1.68352600	-3.46189100	1.40762100
O	0.44724800	-0.67406600	1.30241300
O	2.85585100	-1.93734500	-0.33996800
C	2.44910300	-0.94913700	-1.32172300
C	3.36327100	0.25013400	-1.24074300
O	4.69284800	-0.09696200	-1.67039500
C	2.91908700	1.40161400	-2.17114900
O	2.00960600	2.29050700	-1.56844000
C	4.25722600	2.11817000	-2.44432200

O	4.56216900	2.97774800	-1.35922200
C	5.25944700	0.97413800	-2.46447500
H	1.43054700	-0.62521500	-1.10749300
H	2.49915800	-1.39250800	-2.32146300
H	3.39223900	0.62861300	-0.21042700
H	2.51489700	0.99371300	-3.10811300
H	1.08877800	2.12540700	-1.89757900
H	4.24324600	2.66777200	-3.39631800
H	3.73522800	3.44828400	-1.16759200
H	6.21497600	1.28832100	-2.03347700
O	-1.96279900	-2.46178300	-2.50699100
H	-2.04856200	-1.44528900	-2.53055100
H	-1.60256400	-2.73878200	-3.35717300
C	-4.81459700	-0.68031200	-4.57484800
H	-5.90273100	-0.67655000	-4.71091500
H	-4.51829200	-1.70781800	-4.34343400
H	-4.55554700	-0.05726000	-3.71577600
H	-0.26595900	-5.86543700	3.62724400
H	8.09628600	-2.45150900	5.21267500
H	6.41522800	-2.76846700	4.76128300
H	5.42998600	0.60072200	-3.48100600
H	-5.19096600	3.54819700	-6.57395800
H	-4.28600000	-0.87015300	-6.67577800
H	-2.10842300	4.66463500	2.01324100
H	0.13751800	7.96507000	1.80534000
C	1.50153600	-6.29921300	-1.04714500
H	1.43188800	-7.38371600	-0.91482600
C	2.76518500	-5.79212600	-0.35974800
H	2.66677600	-5.90441000	0.72682900
C	4.01435000	-6.51005400	-0.85500000
H	4.13957800	-6.36308200	-1.93186300
H	4.91963700	-6.15350800	-0.35310500
H	3.92945300	-7.58312200	-0.66228400
O	2.98781100	-4.36362000	-0.62141300
H	4.61133100	-3.70093000	-0.09933900
H	1.54076600	-6.08587500	-2.11949000
H	0.59591300	-5.85162000	-0.63325000

Product 3

C	-12.94700200	-83.34398800	88.03099200
C	-12.32834900	-84.68786400	88.42982900
O	-11.13673600	-84.94270100	88.24285500
C	-12.12983100	-82.10419500	88.45776400
C	-10.83337300	-81.94412200	87.63902500
C	-11.88909600	-82.09990500	89.97400200
C	-10.09533700	-80.62370000	87.88222900
H	-13.04798100	-83.35526600	86.93845000
H	-12.77252600	-81.24286600	88.21821900
H	-11.08862300	-82.01643000	86.57360200
H	-10.17401100	-82.79304100	87.85075600
H	-11.44741800	-81.15436900	90.30082700

H	-12.83041500	-82.23183700	90.52073200
H	-11.21137000	-82.90830000	90.26890200
H	-9.23847400	-80.53120400	87.20802200
H	-10.74866000	-79.76074500	87.70533900
H	-9.71192000	-80.54826800	88.90491800
N	-13.17386000	-85.59214500	88.98864700
C	-12.76599400	-86.94801100	89.35801200
H	-14.15822200	-85.36754400	89.02490600
H	-11.70663800	-86.89465100	89.62066700
C	-1.30700200	-89.13199900	99.58599900
C	-0.94702000	-89.96915500	98.34110600
C	-1.92731300	-89.81576200	97.22592400
N	-1.98058700	-88.71417800	96.39655900
C	-2.98407500	-90.60686800	96.80373800
C	-3.02223100	-88.86381700	95.53120300
N	-3.64827000	-90.00900000	95.76308800
H	-1.36606100	-88.06530900	99.34640100
H	-0.90152600	-91.02899400	98.60982200
H	0.05575200	-89.69720600	97.98755800
H	-1.34307200	-87.93074700	96.41580500
H	-3.28478900	-91.56420100	97.20244700
H	-3.29129000	-88.13761800	94.77121500
C	-9.42000000	-91.88700000	98.14799900
C	-9.87382500	-90.72388400	99.01768900
O	-10.53201600	-90.86299300	100.04028000
C	-10.63330100	-92.60568200	97.50577900
C	-11.32070300	-91.71728700	96.46650500
O	-10.63895600	-91.50753200	95.38545300
O	-12.43314400	-91.23067200	96.71498700
H	-8.71201900	-91.56338100	97.38456000
H	-11.35107200	-92.87608100	98.28229000
H	-10.28094300	-93.51530400	97.00946700
N	-9.51513000	-89.48244300	98.53907600
C	-9.90470100	-88.25947700	99.18061400
C	-8.71259600	-87.37660400	99.58466300
O	-8.81875400	-86.55329700	100.48078500
H	-8.98638600	-89.44852600	97.66998300
H	-10.46723300	-88.50436300	100.08191500
H	-10.50783800	-87.63581700	98.50792200
N	-7.56066700	-87.54970800	98.86512700
C	-6.39378900	-86.74065300	99.09333200
C	-6.41719500	-85.36051400	98.34963800
O	-5.79165500	-84.42611900	98.82485400
C	-5.11809500	-87.50911300	98.71123900
C	-4.96925800	-88.74870200	99.60153200
O	-4.48787800	-88.65922200	100.72530900
N	-5.40812100	-89.91726500	99.06892800
H	-7.58988600	-88.12552100	98.02454300
H	-6.37260400	-86.46918500	100.15156800
H	-4.26339900	-86.85554600	98.89676800
H	-5.13379800	-87.78606900	97.65117100

H	-5.44562600	-90.70891500	99.69637800
H	-5.94112700	-89.95299000	98.20067100
N	-7.12137400	-85.30103000	97.18790500
C	-7.14992200	-84.08374800	96.39667700
C	-8.41976800	-83.22399800	96.54760900
O	-8.43632000	-82.10639600	96.01883200
H	-7.53174300	-86.15414000	96.80091600
H	-7.06148000	-84.31844500	95.33084400
H	-6.30627100	-83.45346700	96.68071300
N	-9.44557800	-83.75842900	97.22942700
C	-10.78499900	-83.17800100	97.22599900
C	-11.63406300	-83.80434800	98.35040600
C	-13.13130800	-83.95903000	97.98777700
C	-13.73036100	-85.29803000	98.43473700
N	-13.12403600	-86.40200000	97.68564200
C	-13.59822500	-87.65156900	97.64398300
N	-14.63504300	-88.01050300	98.43905700
N	-13.09462500	-88.52725500	96.78284400
H	-9.44847800	-84.77219400	97.33865400
H	-11.25300000	-83.37300400	96.25241200
H	-11.52337200	-83.22573600	99.27277300
H	-11.21576400	-84.78997200	98.57184400
H	-13.27115800	-83.87845200	96.90323600
H	-13.72603900	-83.15810700	98.43861600
H	-14.81156100	-85.30212600	98.25475700
H	-13.57068800	-85.43056600	99.51578600
H	-12.21714200	-86.25453400	97.21608500
H	-14.96757500	-88.96174200	98.39572500
H	-14.80508400	-87.51020000	99.29672200
H	-13.16023800	-89.54030500	96.90584200
H	-12.35472900	-88.24533900	96.13125800
C	-9.21500000	-78.87899900	96.13900100
C	-8.55338400	-79.14058300	94.78613200
C	-9.55014200	-79.28851100	93.62704500
N	-10.62481500	-80.27058300	93.83681900
C	-10.57702500	-81.57154600	93.49478300
N	-9.41058500	-82.14213000	93.18435500
N	-11.71227400	-82.27798000	93.43133100
H	-9.86355900	-77.99467700	96.11023600
H	-9.80827500	-79.74177900	96.45291600
H	-7.92829300	-80.03417300	94.86811900
H	-7.88672100	-78.31070000	94.52101700
H	-9.03239000	-79.56461000	92.70365400
H	-10.04974800	-78.33131300	93.44461300
H	-11.46463600	-79.95381000	94.29717800
H	-9.41082200	-83.10265300	92.80559500
H	-8.60419400	-81.87890700	93.73722600
H	-11.69328400	-83.31363600	93.35178900
H	-12.59146500	-81.79816500	93.31765800
Mg	-10.30723800	-89.94770600	94.30208800
P	-10.30701900	-85.75240200	92.95978800

O	-9.29884500	-84.79419800	92.30053300
O	-11.57264400	-85.03081100	93.43108200
O	-10.58698200	-87.00862300	92.12727300
P	-9.86829100	-87.17554100	95.58944500
O	-10.85579600	-88.26037800	95.09329400
O	-10.45962900	-86.33654700	96.71783300
O	-9.46749300	-86.23052800	94.32734800
P	-7.31319800	-89.37525600	95.32402500
O	-8.34244900	-89.78032900	94.28994600
O	-7.18901600	-89.83358900	96.75476400
O	-8.48517800	-87.78044000	96.02026200
O	-6.22824100	-88.28808400	94.87714900
C	-6.44270400	-87.51647900	93.66480700
C	-5.46789700	-86.37074600	93.66979200
O	-4.15298500	-86.83754500	93.36158600
C	-5.76910700	-85.30076000	92.60494000
O	-6.74349100	-84.36692200	93.00874000
C	-4.39118800	-84.61957800	92.48407500
O	-4.23860200	-83.67394600	93.53007700
C	-3.41600200	-85.77500100	92.72200000
H	-7.46215300	-87.12908500	93.66446600
H	-6.27840200	-88.16190300	92.79725900
H	-5.48007100	-85.88537200	94.65521400
H	-6.03732700	-85.78029300	91.65296000
H	-7.64305600	-84.63232100	92.69237100
H	-4.24443500	-84.15026900	91.50140600
H	-5.07143100	-83.17659300	93.55384100
H	-2.59371400	-85.43722700	93.36231700
O	-10.89614400	-89.52568000	92.43305500
H	-10.80700500	-88.51334800	92.30938100
H	-10.58002700	-89.94444600	91.62437300
C	-13.59018000	-87.45585000	90.53760700
H	-14.65941100	-87.49639000	90.29731700
H	-13.27424600	-88.46781300	90.80589100
H	-13.44282100	-86.81700800	91.41191900
H	-8.91410200	-92.59455400	98.81325300
H	-0.53478600	-89.25463800	100.35090600
H	-2.26281400	-89.43218600	100.01692600
H	-3.00206400	-86.16751500	91.78702100
H	-13.96021300	-83.27658600	88.44561400
H	-12.86406100	-87.61774900	88.49268400
H	-10.69106300	-82.09468600	97.33531200
H	-8.45956500	-78.70903900	96.91047300
C	-7.40011700	-92.70535800	94.25949800
H	-7.40012900	-93.79732200	94.33531300
C	-6.09603100	-92.17933300	94.84079400
H	-6.07873300	-92.34954100	95.92509600
C	-4.88215600	-92.83229000	94.18321300
H	-4.89921200	-92.66062200	93.10276100
H	-3.94168300	-92.44022300	94.57790500
H	-4.90273900	-93.91183600	94.36024200

O	-5.98676900	-90.73000300	94.63015400
H	-5.08666800	-90.44263300	95.01140300
H	-7.47783800	-92.43716900	93.20161400
H	-8.28007300	-92.33068300	94.78285400
